# The tarantula toxin *β*/*δ-TRTX-Pre1a* highlights the importance of the S1-S2 voltage-sensor region for sodium channel subtype selectivity

**DOI:** 10.1038/s41598-017-01129-0

**Published:** 2017-04-20

**Authors:** Joshua S. Wingerd, Christine A. Mozar, Christine A. Ussing, Swetha S. Murali, Yanni K.-Y. Chin, Ben Cristofori-Armstrong, Thomas Durek, John Gilchrist, Christopher W. Vaughan, Frank Bosmans, David J. Adams, Richard J. Lewis, Paul F. Alewood, Mehdi Mobli, Macdonald J. Christie, Lachlan D. Rash

**Affiliations:** 1 Institute for Molecular Bioscience, The University of Queensland, St Lucia, QLD 4072 Australia; 2 Centre for Advanced Imaging & School of Chemistry and Molecular Biosciences, The University of Queensland, St Lucia, QLD 4072 Australia; 3 School of Biomedical Sciences, The University of Queensland, St Lucia, 4072 QLD Australia; 4 Discipline of Pharmacology, University of Sydney, Camperdown, NSW 2006 Australia; 5 Pain Management Research Institute, University of Sydney, St Leonards, NSW 2006 Australia; 6 Illawarra Health and Medical Research Institute, University of Wollongong, Wollongong, NSW 2522 Australia; 7 Department of Physiology and Solomon H. Snyder Department of Neuroscience, Johns Hopkins University School of Medicine, Baltimore, Maryland 21205 USA; 8 Novo Nordisk A/S, Copenhagen Area, Capital Region, Denmark; 9 Harvard Medical School, Children’s Hospital, 300 Longwood Ave, Boston, MA 02115 United States

## Abstract

Voltage-gated sodium (Na_V_) channels are essential for the transmission of pain signals in humans making them prime targets for the development of new analgesics. Spider venoms are a rich source of peptide modulators useful to study ion channel structure and function. Here we describe β/δ-TRTX-Pre1a, a 35-residue tarantula peptide that selectively interacts with neuronal Na_V_ channels inhibiting peak current of hNa_V_1.1, rNa_V_1.2, hNa_V_1.6, and hNa_V_1.7 while concurrently inhibiting fast inactivation of hNa_V_1.1 and rNa_V_1.3. The DII and DIV S3-S4 loops of Na_V_ channel voltage sensors are important for the interaction of Pre1a with Na_V_ channels but cannot account for its unique subtype selectivity. Through analysis of the binding regions we ascertained that the variability of the S1-S2 loops between Na_V_ channels contributes substantially to the selectivity profile observed for Pre1a, particularly with regards to fast inactivation. A serine residue on the DIV S2 helix was found to be sufficient to explain Pre1a’s potent and selective inhibitory effect on the fast inactivation process of Na_V_1.1 and 1.3. This work highlights that interactions with both S1-S2 *and* S3-S4 of Na_V_ channels may be necessary for functional modulation, and that targeting the diverse S1-S2 region within voltage-sensing domains provides an avenue to develop subtype selective tools.

## Introduction

Voltage-gated sodium channels (Na_V_s) are membrane proteins with four homologous domains (DI-DIV), each composed of six transmembrane segments (S1-S6) assembled into a single, functional α-subunit (~260 kDa). Each domain has two functionally distinct regions; the S1-S4 segments comprise the voltage-sensing domain (VSD) whereas the S5-S6 helices and extracellular ‘P-loop’ form the selectivity filter and ion-conducting pore. There are nine mammalian α-subunits (Na_V_1.1 to Na_V_1.9) with greater than 64% sequence identity between isoforms 1.1 to 1.7, whereas 1.8 and 1.9 share ~50–60% identity with other members^1, 2^. The Na_V_ channel family contains important therapeutic targets for local anaesthetics, anti-arrhythmics, analgesics, and anti-epileptics^3, 4^. For example, the peripherally expressed neuronal Na_V_1.7 has been identified as a potential target for the treatment of chronic pain, largely from studies of human channelopathies^5^. Mutations of *SCN9A* that result in Na_V_1.7 gain-of-function underlie paroxysmal extreme pain disorder (PEPD) and primary erythromelalgia^6, 7^, whereas mutations that result in the loss of Na_V_1.7 function underlie ‘congenital insensitivity to pain’ (CIP)^8^. The neuronal isoforms Na_V_1.3, Na_V_1.8 and Na_V_1.9 have also been implicated in various forms of chronic and acute pain^9–12^, whereas recent evidence suggests that Na_V_1.1 also plays a role in mechanical pain transmission^13^. However, the similarity between Na_V_ channel α-subunits poses a challenge to discover and develop molecules that can selectively modify the function of each subtype.

Natural toxins have been instrumental in defining Na_V_ channel subtypes and many of the known functionally relevant binding sites on the channel^1^. In recent decades, venom peptides have proven to be an invaluable source of novel, selective, and potent modulators of ion channels, leading to an increasing interest in these molecules as pharmacological tools and potential therapeutic leads^14^. In this respect, spiders possibly represent one of the richest sources of novel voltage-gated channel modulators^15^ that not only target channel α-subunits, but also complexes containing the accessory β-subunits^16^. A majority of currently characterised Na_V_ channel modulating spider venom peptides share a common structural motif of the inhibitory cysteine knot (ICK) and interact with one of four VSDs at partially defined receptor sites^17, 18^.

These VSD modulating peptides have been described to modify channel gating in three distinct ways depending on the domain they target and the effect on that domain^17^. Domain IV of the Na_V_ channel α-subunit uniquely controls inactivation of the channel^19^, thus spider peptides that interact with and hinder the normal function of VSDIV slow or inhibit fast inactivation of the channel and may result in persistent current^20^. Conversely, spider venom peptides that interact with DI, DII, and DIII VSD modulate channel activation^21^. Most of those discovered to date cause a shift in the voltage-dependence of activation in the depolarising direction and inhibit activation^22, 23^, whereas some such as β-HXTX-Mg1a (Magi 5) cause a hyperpolarising shift, thereby facilitating activation^24^. The VSDs of different voltage-gated ion channels exhibit higher sequence variability than the highly conserved pore region, thus offering the opportunity for subtype-selective interactions between Na_V_ subtypes as well as the greater voltage-gated ion channel superfamily. Indeed, certain families of spider venom peptides demonstrate remarkable subtype selectivity profiles^13, 25^.

A deeper understanding of the molecular basis of the interactions between spider venom peptides with Na_V_ channels is helping efforts to design a new generation of pharmacological tools and potential therapeutic lead molecules^26–28^. To this end, we identified β/δ-TRTX-Pre1a (Pre1a) from the venom of the tarantula *Psalmopoeus reduncus* in a screen for Na_V_1.7 inhibitors. Pre1a exhibited a unique and complex pharmacological profile across neuronal Na_V_ channel subtypes where it preferentially inhibits *fast inactivation* of Na_V_1.3, inhibits *activation* of Na_V_1.2, Na_V_1.6, and Na_V_1.7, and inhibits *both* activation and fast inactivation of Na_V_1.1, with no effect on Na_V_1.4 or Na_V_1.5 at sub-micromolar concentrations. This functional profile is dictated by classical interactions with the DII and DIV S3-S4 loops of the Na_V_ channel VSDs. However, our evidence points to the S1-S2 loops as critical for imparting the isoform selectivity demonstrated by Pre1a. Pre1a thus represents a valuable tool to study the subtle differences in DII and DIV interaction sites between members of the Na_V_ channel family.

## Results

### Isolation and sequence of TRTX-Pre1a

**Figure 1:**
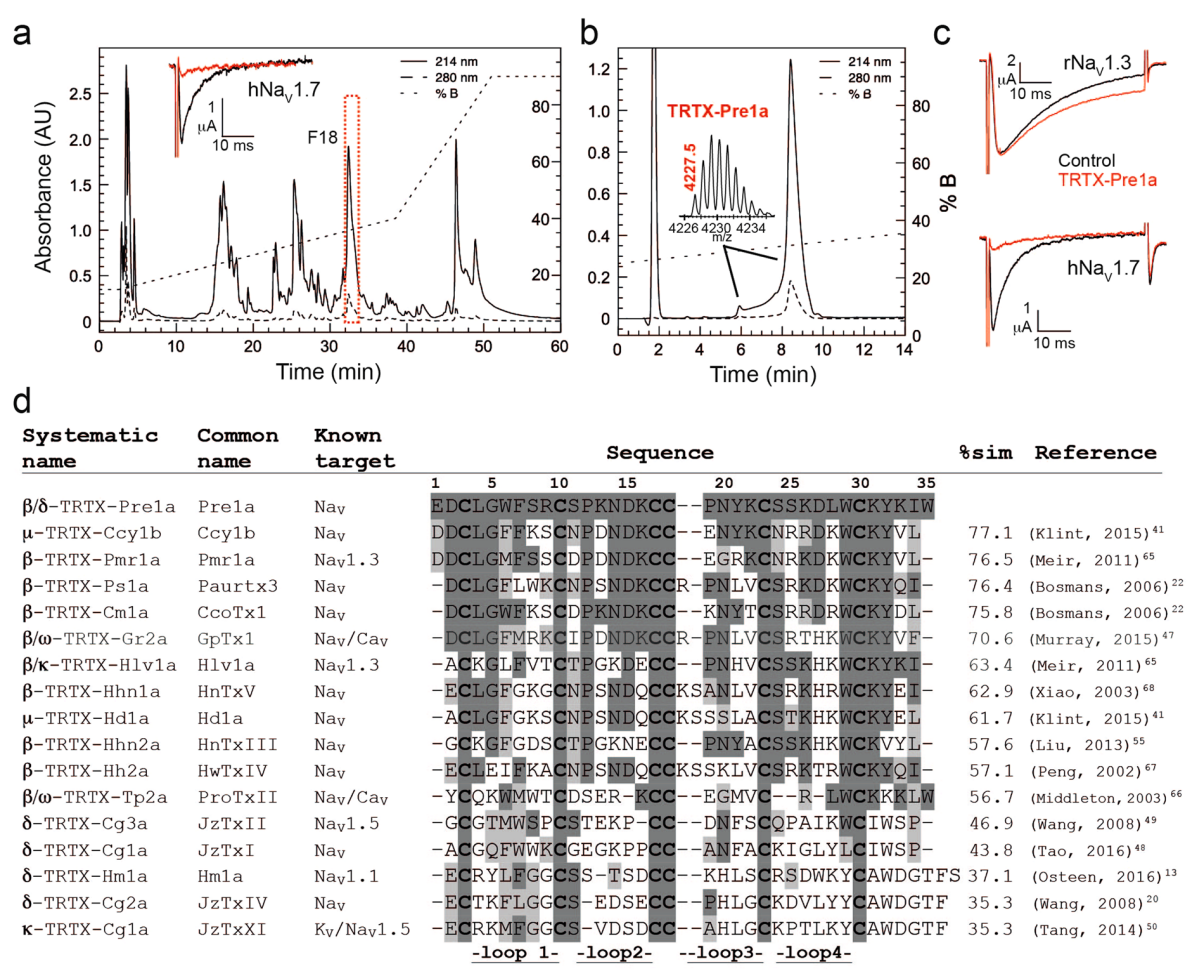
Identification and sequence of TRTX-Pre1a. (**a)** RP-HPLC chromatogram of crude venom from *P. reduncus* indicating the fraction (F18) responsible for robust inhibition of hNa_V_1.7 expressed in *Xenopus* oocytes (inset). (**b)** Final analytical RP-HPLC purification step of F18 (TRTX-Pre1a) (inset: MALDI-TOF MS spectrum showing M + H^+^ of 4227.5). (**c)** Activity of pure, native Pre1a on rNa_V_1.3 and hNa_V_1.7 exressed in *Xenopus* oocytes demonstrating inhibition of inactivation and peak current, respectively. (**d)** Sequence alignment of TRTX-Pre1a with Na_V_ modulating Theraphotoxins^13, 20, 22, 41, 47–50, 55, 65–68^. Percent similarity was calculated comparing the number of identical (dark gray) and similar (light gray) amino acids.

In a small screen of 14 spider venoms, *Psalmopoeus reduncus* venom at 1:1000 dilution (~200 μg/ml) consistently and potently inhibited human (h) Na_V_1.7 expressed in *Xenopus laevis* oocytes. Activity-guided fractionation resulted in the identification of a major component exhibiting inhibitory activity at hNa_V_1.7 expressed in oocytes (Fig. 1a). The purified peptide eluted with an unusual, leading minor peak that upon mass analysis revealed the same M + H^+^ of 4227.5 as the principal peak (Fig. 1b) and was found to inhibit both the of peak current of hNa_V_1.7 and the inactivation of rat (r) Na_V_1.3 (Fig. 1c). The mass of the reduced/alkylated peptide was 588 Da higher than the native peptide (6 × 98, the MW of maleimide), indicating the presence of three disulfide bonds (data not shown). Edman analysis of the reduced/alkylated peptide resulted in complete sequence determination of a novel 35 amino acid peptide with high sequence similarity to several Na_V_ modulating spider venom peptides (Fig. 1d), determined by a search of non-redundant protein database (NCBI). Sequences were aligned and percent similarity calculated manually. Taking into account disulfide bonds, the observed monoisotopic M + H^+^ (4227.5) matched that of the theoretical M + H^+^ with a free acid C-terminus (4226.9, Expasy-PeptideMass) that was confirmed using MSMS on tryptic digest fragments (data not shown). The peptide was named TRTX-Pre1a (Pre1a) according to the current rational nomenclature^29^.

**Figure 2:**
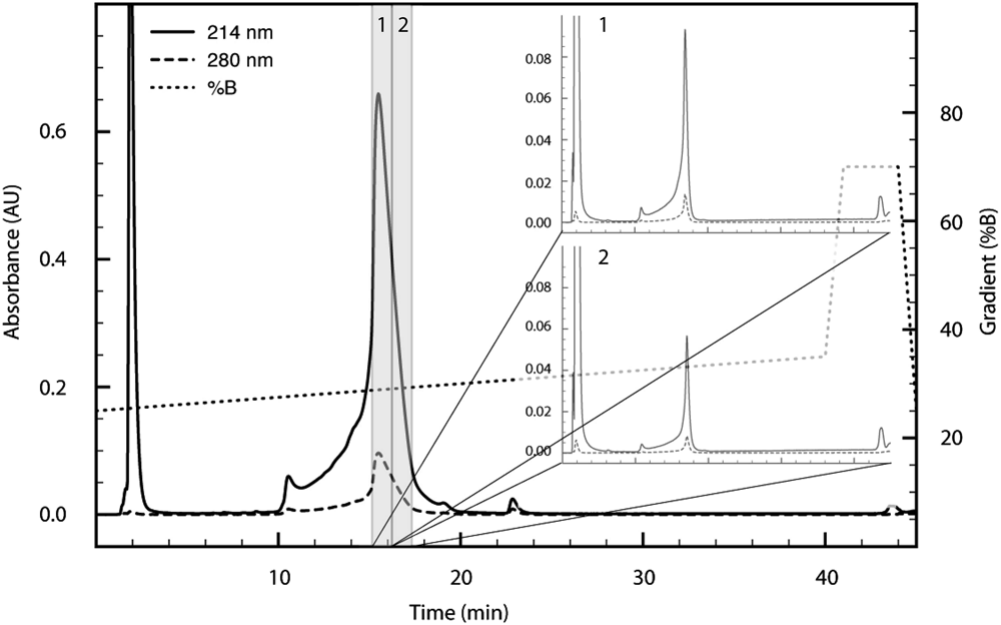
Pre1a shows conformational flexibility under RP-HPLC conditions. Analytical RP-HPLC of pure synthetic Pre1a shows the presence of multiple conformers in and acetonitrile/water mixture at room temperature. Insets 1 and 2, demonstrate identical elution profiles for reinjection of two fractions (highlighted and numbered) taken from the major peak, discounting the presence of impurities.

In order to carry out functional characterisations, we produced Pre1a using Boc solid-phase peptide synthesis. The folded synthetic peptide co-eluted with the peptide isolated from the native source (Supp. Fig. 1). Analytical RP-HPLC analysis of synthetic Pre1a revealed that the unusual non-symmetrical nature of the eluting peptide is an inherent property of the peptide and suggested that Pre1a is structurally heterogeneous (Fig. 2). This structural heterogeneity was confirmed by collecting the body and trailing portions of the major peak as individual fractions then re-injecting under the same conditions, whereupon the same non-symmetrical chromatographic profile was observed (Fig. 2 insets). This suggests a conformational (chemical) exchange process that is slow on the HPLC time scale.

### Structural studies of recombinant Pre1a

Preliminary homonuclear NMR analysis of synthetic Pre1a confirmed the presence of conformational heterogeneity, prompting us to isotope label the peptide (using recombinant expression in *E. coli*, Supp. Fig. 2) to enable less ambiguous heteronculear NMR analysis. Backbone resonance assignments (^1^H_N_, ^15^N, ^13^C_α_, ^13^C_β_, ^13^C′) for rPre1a were completed by analysis of the 3D HNCACB, CBCA(CO)NH, and HNCO spectra, and the side chain ^1^H and ^13^C chemical shifts from the 3D H(CC) (CO)NH-TOCSY and (H)CC(CO)NH-TOCSY spectra, respectively. Together we achieved 90% completion of all proton assignments. However, due to the structural heterogeneity, insufficient unique constraints were derived from the NOESY spectra to enable the structure calculations using CYANA^30^ to produce a adequately converged high-resolution structure. However, the chemical shifts determined for the backbone resonances allowed us to predict the secondary structure of Pre1a using the program TALOS+ ^31^, which suggests that residues around C3–L4 and Y21–K22 are likely to form β-strands (Supp. Fig. 3a).

Furthermore, the NMR data allowed us to confidently determine the disulfide connectivity of the peptide via unambiguous NOEs between sidechain C_β_ filtered H_β_ protons. NOEs could be observed between the side chain protons of C3 and C18, C10 and C23 and C17 and C30 in the ^13^C-NOESY (Supp. Fig. 3b), consistent with the formation of the common ICK-motif (C1–C4, C2–C5, C3–C6), which appears to be the dominant structural scaffold among Na_V_ channel modulating spider venom peptides characterised to date.

**Figure 3:**
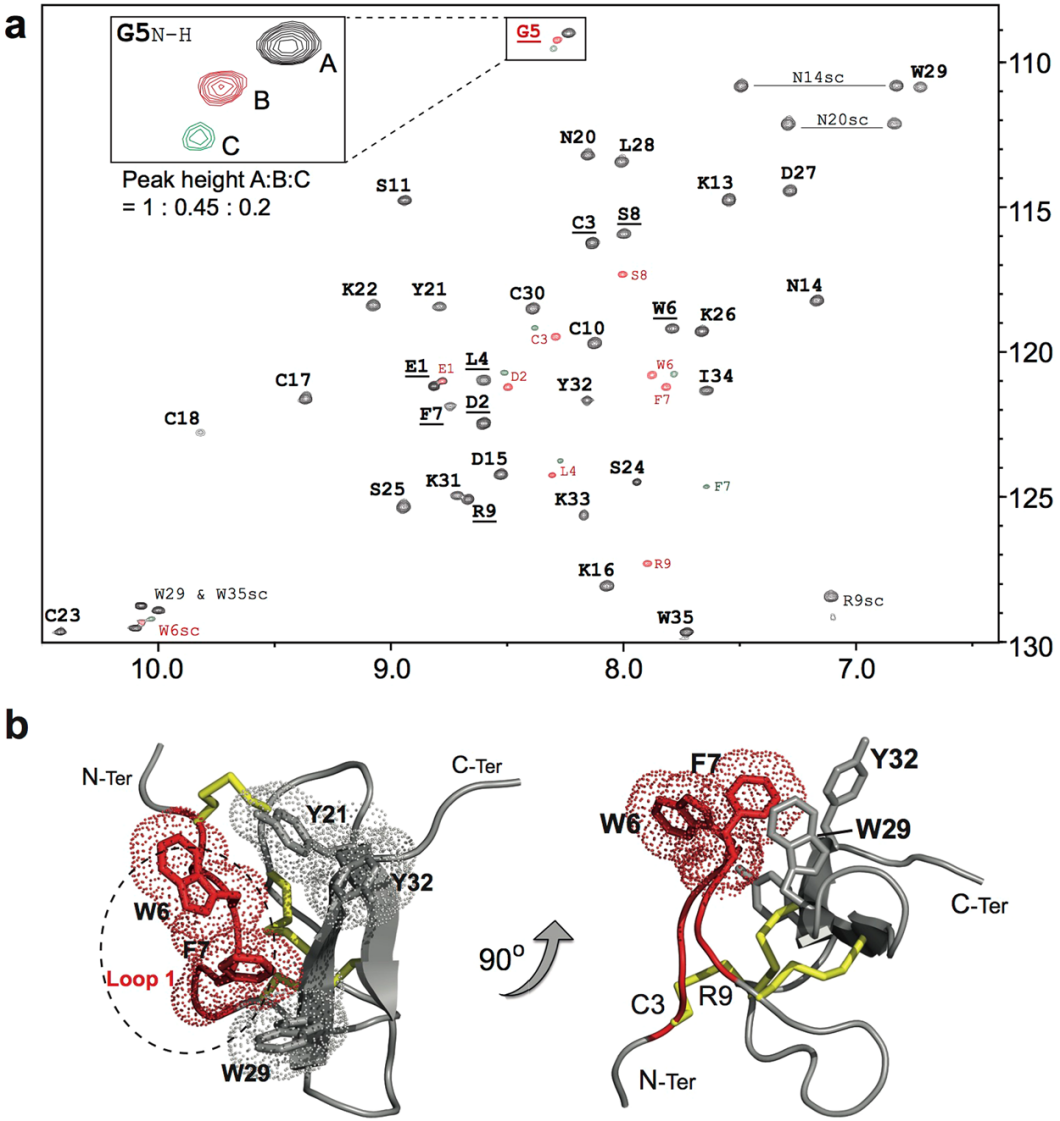
Pre1a shows conformational flexibility in aqueous conditions. (**a)** 2D ^1^H-^15^N-HSQC of recombinantly produced Pre1a. The chemical shifts of resonances for residues in loop 1 (D2–R9, underlined) show multiple peaks indicating the presence of three conformations of the peptide (A = Major, B = middle, C = minor, highlighted for G5 in the inset). sc = side chain NH resonances for N, R and W residues. (**b)** Two views of a homology model of Pre1a (based on the NMR structure of HwTxIV, PDB: 2M4X) illustrating the relative positions of W6 and F7 at the tip of Loop 1 and W29, Y32 and Y21. Loop 1 residues (that show multiple peaks in the HSQC above) are in red, disulfide bonds are in yellow. The right panel highlights the position of W6 and F7 at the tip of Loop 1 and the positions of C3 and R9, which may act as a hinge region for movement of the loop.

The 3D NMR data also allowed us to characterise the conformational heterogeneity of Pre1a, first observed on RP-HPLC. The presence of several peptide conformations resulted in multiple backbone ^1^H_N_/^15^N chemical shifts for residues D2–R9 in the ^15^N-HSQC spectrum, exemplified by G5 (Fig. 3a). These residues form Loop 1 and, with the exception of S8 and R9 (which have duplicate peaks), all have visible triplicate peaks with decreasing intensities (for examples of NMR experiments on related but structurally rigid peptides see Fig. 2 in Lau *et al*.^32^ and Fig. S3 in Klint *et al*.^33^). Additionally, a homology model of Pre1a was constructed using the structure of HwTx-IV as a template (PDB 2M4X)^34^ using SWISS-MODEL^35^. The Pre1a model shows a highly dense packing of five aromatic residues with W6 and F7 at the tip of Loop 1 falling between W29 and Y32/Y21 on one face of the peptide (Fig. 3b). We hypothesise that this overcrowding of bulky aromatic side chains results in the structural flexibility of Pre1a as seen in both aqueous (NMR) and hydrophobic (RP-HPLC) conditions.

### Pre1a affects neuronal Na_V_ channels in a subtype-dependent manner

**Figure 4:**
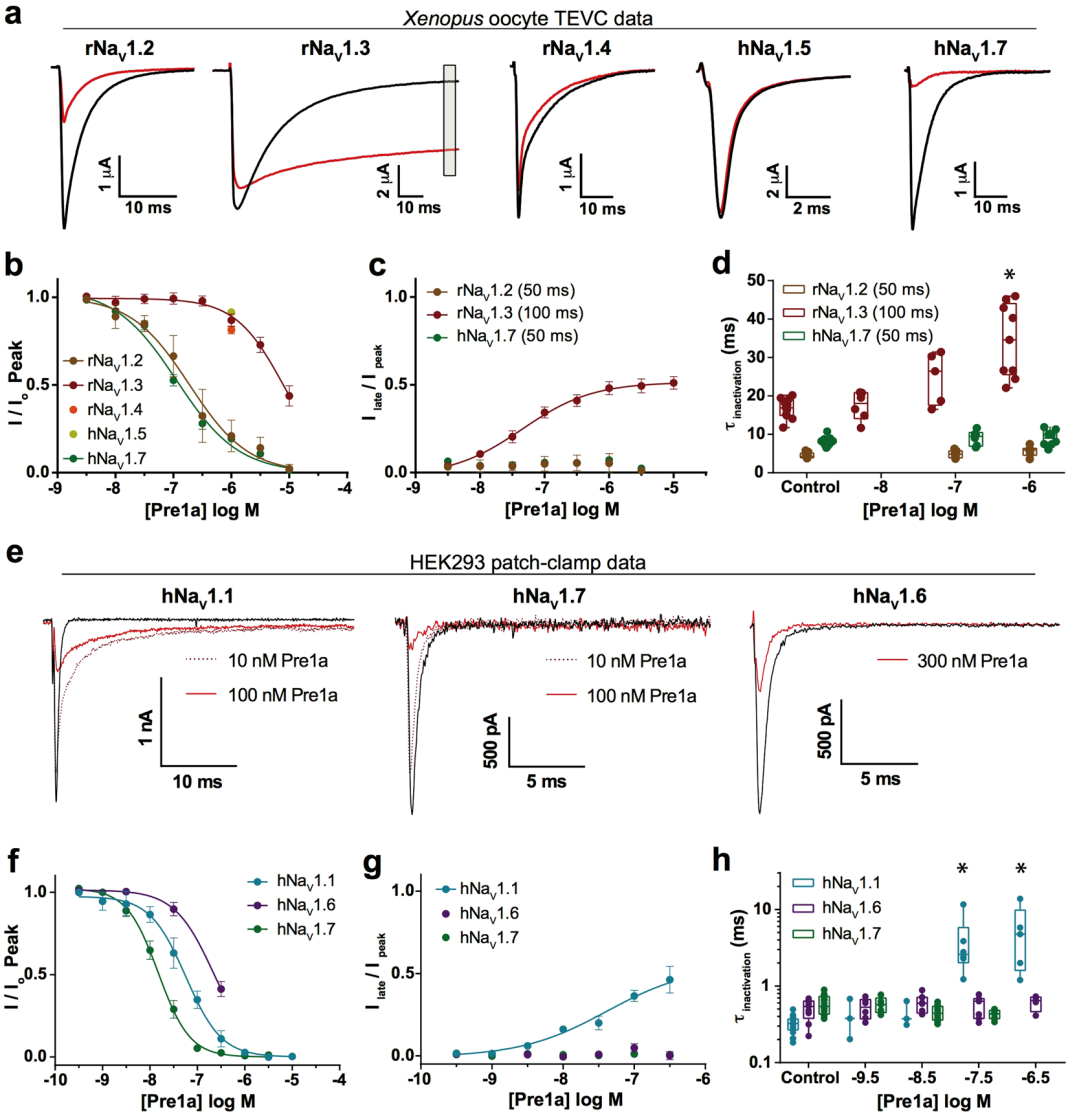
Pre1a preferentially inhibits neuronal Na_V_ channels. (**a)** Representative Na_V_ currents recorded from *Xenopus* oocytes (α-subunit alone) before addition of 1 µM Pre1a (black) and after reaching steady state inhibition (red). Late current was assessed at 50 ms (100 ms for Na_V_1.3) from the peak current, as highlighted by the grey box on rNa_V_1.3. **(b)** Concentration-effect curves for peak current inhibition by Pre1a for rNa_V_1.2 (IC_50_ 189.6 nM; n ≥ 5), rNa_V_1.3 (IC_50_ 8.0 μM; n ≥ 8) and hNa_V_1.7 (IC_50_ 114.0 nM; n ≥ 8), with single point 1 µM concentrations for rNa_V_1.4 (n = 6) and hNa_V_1.5 (n = 7). **(c)** Concentration-response curve for late current inhibition of inactivation by Pre1a of rNa_V_1.3 (EC_50_ 45.0 nM). **(d)** Concentration-dependent effects of Pre1a on the rate of inactivation (τ) for rNa_V_1.3, with 1 µM demonstrating a significant slowing of inactivation (p < 0.001; n ≥ 5; ANOVA with Dunnet’s test). **(e)** Representative current traces of hNa_V_1.1, hNa_V_1.6, and hNa_V_1.7 expressed in HEK cells co-expressed with Na_V_β1, in the absence (black) and presence (red) of varying Pre1a concentrations. **(f)** Concentration-response curves for peak current inhibition by Pre1a for hNa_V_1.1 (IC_50_ 57.1 nM; n ≥ 6), hNa_V_1.6 (IC_50_ 221.6 nM; n ≥ 6), and hNa_V_1.7 (IC_50_ 15.0 nM; n ≥ 9) expressed in HEK cells. **(g)** Concentration-response curves for Pre1a effects on late current (measured at 10 ms from peak) of hNa_V_1.1 (EC_50_ 41.4 nM), with no measurable effect on hNa_V_1.6 or hNa_V_1.7. **(h)** Concentration-dependent effects of Pre1a on the rate of inactivation (τ) for hNa_V_1.1, with 30 nM and 300 nM demonstrating a significant slowing of inactivation (p < 0.001; n ≥ 5; ANOVA with Dunnet’s test).

Pre1a produced by either chemical synthesis or recombinant expression was equipotent at inhibiting the peak current of hNa_V_1.7 (Supp. Fig. 4a). β-subunits can have profound effects on venom peptide interactions with Na_V_ channels^36^, however Pre1a’s inhibitory effect on hNa_V_1.7 was not modified by the accessory β1 subunit (Supp. Fig. 4b). To determine the subtype selectivity profile of recombinant Pre1a it was tested on five different Na_V_ channel α-subunits expressed in *X. laevis* oocytes (Fig. 4a). Pre1a concentration-dependently inhibited the peak inward current of rNa_V_1.2 and hNa_V_1.7 with IC_50_ values of 189.6 nM (pIC_50_ 6.72 ± 0.06) and 114.0 nM (pIC_50_ 6.94 ± 0.06), respectively (Fig. 4b), and weakly inhibited rNa_V_1.3 with an IC_50_ of 8.0 μM (pIC_50_ 5.10 ± 0.05). Similar to its effect on Na_V_1.3, 1 μM Pre1a only weakly inhibited the peak current amplitude of rNa_V_1.4 and hNa_V_1.5 by 16.47 ± 0.05% and 8.60 ± 0.05%, respectively (Fig. 4b).

Pre1a demonstrated inhibitory effects on rNa_V_1.3 fast inactivation (Fig. 4a), thus these effects on Na_V_1.2, 1.3 and 1.7 were examined in detail. We analysed the rate of fast inactivation (defined as tau, τ_inact_, or the time to reach 36.8% of a single exponential fit of the current decay) and the degree of persistent sodium current measured at 50 ms of depolarisation (Na_V_1.2 and 1.7) and 100 ms for rNa_V_1.3 due to the longer time required to reach complete inactivation in the oocyte expression system without β1. Consistent with results observed during assay-guided fractionation, Pre1a potently inhibited the inactivation of rNa_V_1.3 with an EC_50_ of 45.5 nM (pEC_50_ 7.34 ± 0.27) (Fig. 4a and c), a selectivity of more than 150-fold for inhibiting rNa_V_1.3 inactivation over activation. In addition to inhibiting the *extent* of rNa_V_1.3 inactivation (as indicated by the level of persistent current), Pre1a also decreased the *rate* of current inactivation, reflected as a concentration-dependent increase in τ_inact_ (Fig. 4d). At 1 μM, a concentration that almost completely inhibited rNa_V_1.2 and hNa_V_1.7 currents, Pre1a had no significant effect on the persistent current or τ_inact_ of either of these channels. There was no apparent effect of 1 μM Pre1a on the inactivation of either rNa_V_1.4 or hNa_V_1.5 (Fig. 4a).

Spider venom peptides similar to Pre1a have been shown to target Na_V_1.1 and Na_V_1.6^22, 28^, thus we assessed the activity of Pre1a on hNa_V_1.1 and hNa_V_1.6 stably expressed in HEK cells using the Qpatch16 automated patch-clamp platform (Fig. 4e). To compare possible discrepancies in potency between the two different systems, HEK-hNa_V_1.7 cells were tested in parallel. Pre1a inhibited hNa_V_1.1, hNa_V_1.6, and hNa_V_1.7 with IC_50_s of 57.1 nM (pIC_50_ 7.24 ± 0.07), 221.6 nM (pIC_50_ 6.65 ± 0.06), and 15.0 nM (pIC_50_ 7.82 ± 0.04), respectively (Fig. 4f). An approximate 7-fold increase in potency was determined for the inhibition of hNa_V_1.7 peak current as analysed on the QPatch as compared to the IC_50_ obtained for this channel in oocytes. A difference in potency of some molecules or with some receptors is not uncommon when comparing two-electrode voltage clamp of oocytes to data obtained using whole-cell patch clamping of mammalian cells^37–39^.

In addition to the effect of Pre1a on Na_V_1.1 peak current, it also equipotently inhibited Na_V_1.1 fast inactivation with an EC_50_ of 41.4 nM (pEC_50_ 7.38 ± 0.51) (Fig. 4g). Beyond 1 µM, inhibition of peak current prevented the analysis of inactivation as the current was fully inhibited. Concentrations up to 1 μM Pre1a on hNa_V_1.6 or up to 300 nM on hNa_V_1.7 had no effect on inactivation (Fig. 4g). Together, these results demonstrate that Pre1a has a strong preference for modulating neuronal Na_V_ channel isoforms over the skeletal muscle (Na_V_1.4) and the cardiac (Na_V_1.5) isoforms. Using the IC_50_ for inhibition of Na_V_1.7 peak current as a reference point (to allow comparison across platforms) results in a relative rank order potency for Na_V_ modulation of rNa_V_1.3_inact_ (0.4) > hNa_V_1.7_act_ (1) > rNa_V_1.2_act_ (1.7) > hNa_V_1.1_inact_ (2.7) > hNa_V_1.1_act_ (3.8) > hNa_V_1.6_act_ (15) > rNa_V_1.3_act_ (70). Thus the effect of Pre1a on Na_V_1.3 inactivation has the highest relative potency for all Na_V_ activity (peak current or inactivation) on the channels tested.

### Pre1a modulates Na_V_ channel function by inhibiting channel gating

**Figure 5:**
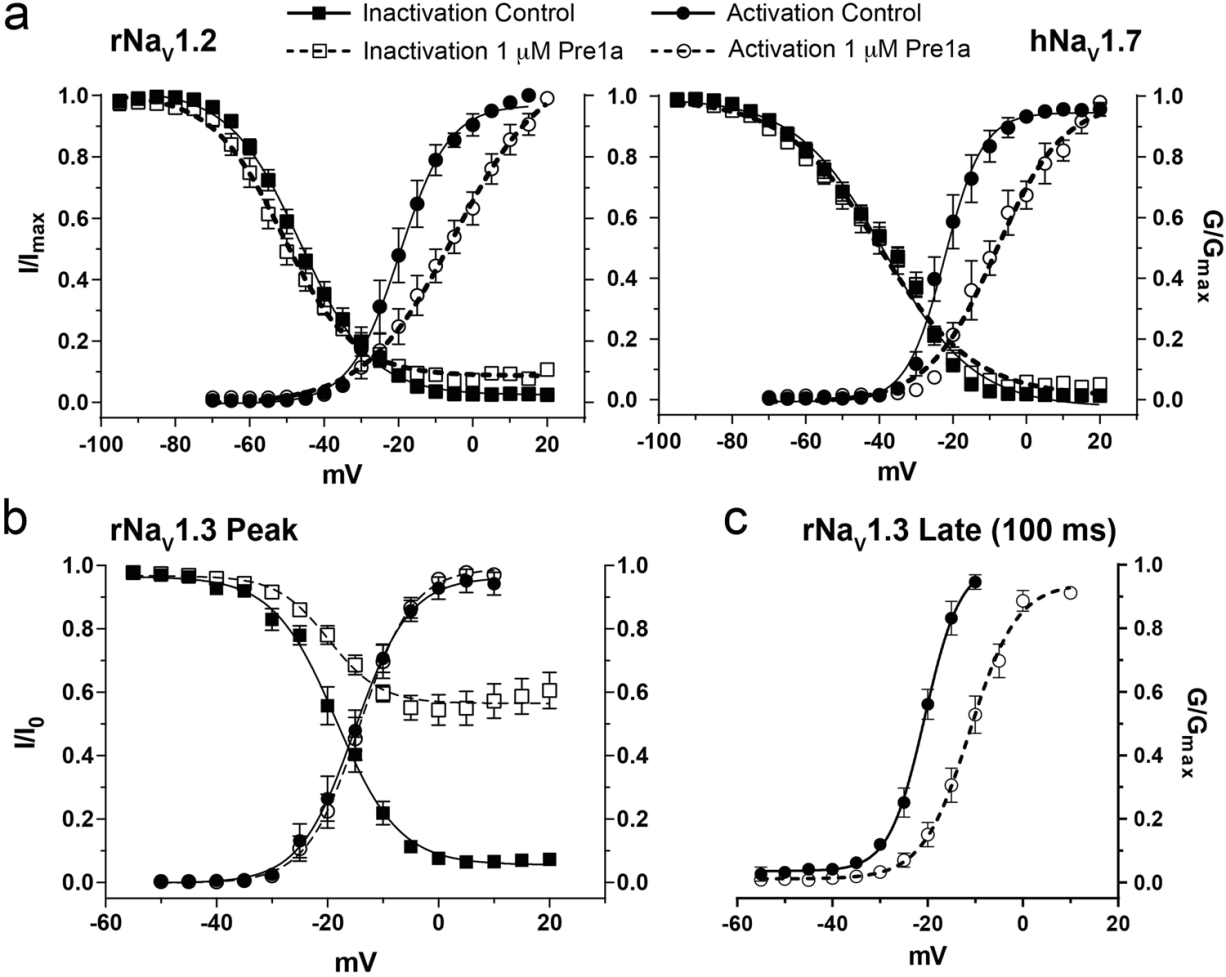
Pre1a (1 μM) affects the voltage-dependence of activation of rNa_V_1.2 and hNa_V_1.7 and the steady-state inactivation (SSIN) of rNa_V_1.3. (**a**) 1 µM Pre1a causes a depolarizing shift in the *V*_*1/2*_ of activation of rNa_V_1.2 (control *V*_*1/2*_ = −19.73 ± 0.85; Pre1a *V*_*1/2*_ = −4.88 ± 2.06, n = 5) and hNa_V_1.7 (control *V*_*1/2*_ = −22.19 ± 0.01, Pre1a *V*_*1/2*_ = −8.37 ± 0.02, n = 6). 1 µM Pre1a had no significant effect on *V*_*1/2*_ inactivation of rNa_V_1.2 (control *V*_*1/2*_ = −45.23 ± 0.37; Pre1a *V*_*1/2*_ = −47.92 ± 0.58) and hNa_V_1.7 (control *V*_*1/2*_ = −38.42 ± 0.61; Pre1a *V*_*1/2*_ = −39.97 ± 0.82). (**b**) Voltage-dependence of activation and SSIN of rNa_V_1.3 in the absence and presence of 1 µM Pre1a (n = 11). Pre1a had no significant effect on voltage-dependence of peak current activation (control *V*_*1/2*_ = −15.13 ± 0.65; Pre1a *V*_*1/2*_ = −14.23 ± 0.52), or SSIN (control *V*_*1/2*_ = −18.41 ± 0.52; Pre1a *V*_*1/2*_ = −19.8 ± 1.26), other than preventing the current from fully inactivating at positive potentials. (**c**) Pre1a (1 μM) caused a strong positive shift in the voltage-dependence of activation for rNa_V_1.3 late current (analysed at 100 ms) (control *V*_*1/2*_ = −20.64 ± 0.54; Pre1a *V*_*1/2*_ = −11.14 ± 0.58).

The mechanism of action of Pre1a was studied by determining the voltage of half maximal effect (*V*_*1/2*_) of channel gating for rNa_V_1.2, rNa_V_1.3 and hNa_V_1.7 expressed in oocytes (Fig. 5). Pre1a (1 µM) significantly shifted the *V*_*1/2*_ of rNa_V_1.2 and hNa_V_1.7 activation by +14.9 mV and +13.8 mV, respectively (Fig. 5a), whilst having no effect on the *V*_*1/2*_ of activation of rNa_V_1.3 peak current (Fig. 5b). Pre1a had no effect on the *V*_*1/2*_ of inactivation of rNa_V_1.2 or hNa_V_1.7 (Fig. 5a) or rNa_V_1.3, however, it did prevent the current of Na_V_1.3 from fully inactivating (Fig. 5b). In contrast, 1 μM Pre1a shifted the *V*_*1/2*_ of activation of the rNa_V_1.3 late current by +9.6 mV (Fig. 5c).

Depolarising shifts in the activation of rNa_V_1.2 and hNa_V_1.7 and of the rNa_V_1.3 late current are consistent with inhibition of channel gating via interactions with the voltage-sensing domains of repeat II and IV, respectively. Therefore, Pre1a is clearly a gating modifier that interacts with Na_V_ channels in a subtype-dependent manner. As Pre1a inhibits both Na_V_ channel activation and fast inactivation, we propose using the prefix β/δ according to the proposed nomenclature for spider venom peptides^29^.

### Pre1a interacts with the S3-S4 linkers of hNa_V_1.7 DII and DIV

**Figure 6:**
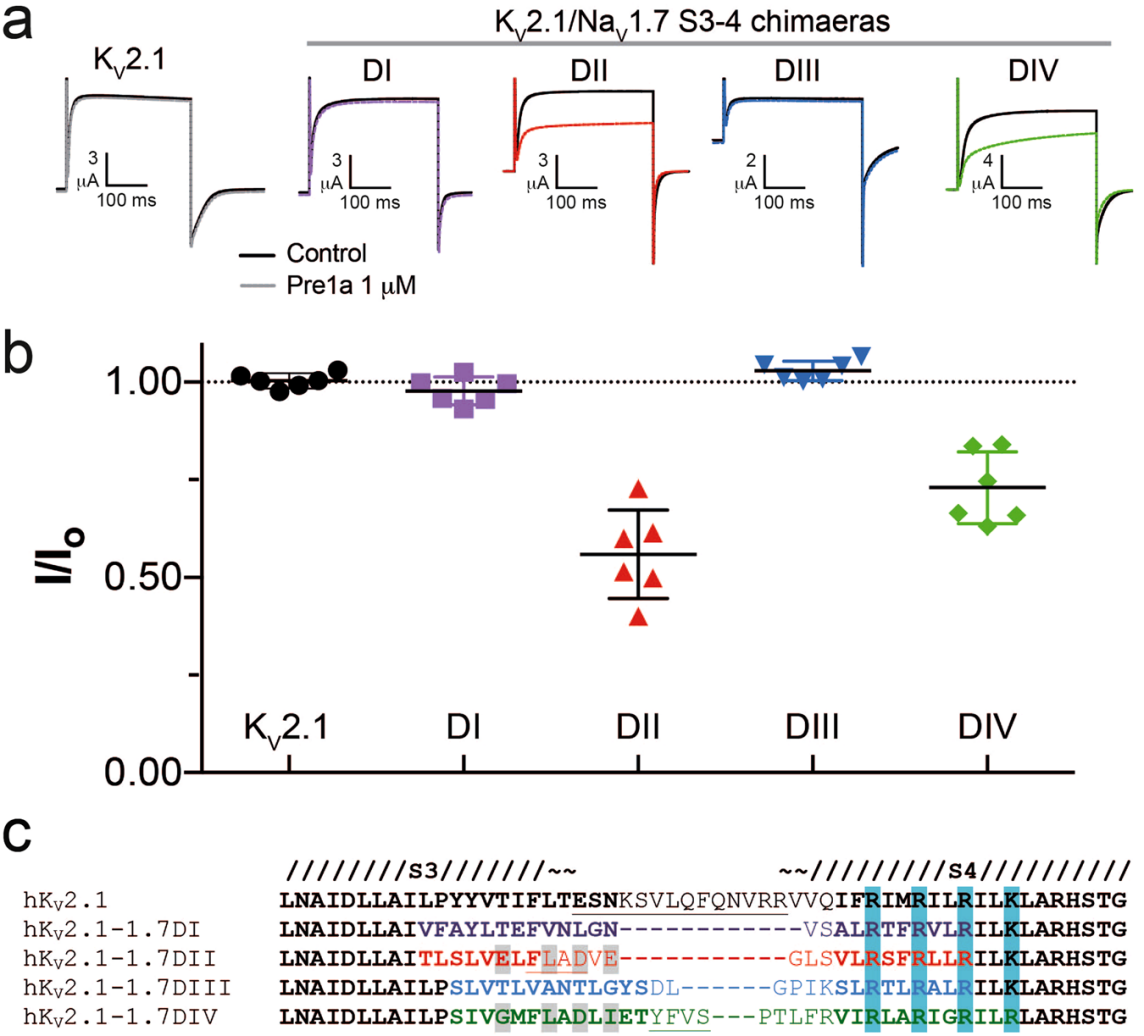
Pre1a can interact with the DII and DIV S3-S4 linker of hNa_V_1.7. **(a)** Representative traces showing the effect of Pre1a (1 µM) on K_V_2.1 and chimaeras of K_V_2.1 containing S3-S4 linker region from each domain of hNa_V_1.7. **(b)** Normalised peak-current inhibition by Pre1a (1 µM) for each K_V_2.1/hNa_V_1.7 chimaera (n = 6). Chimaeras of K_V_2.1 with the hNa_V_1.7 DII and DIV had peak current inhibited after addition of 1 µM Pre1a by 44.0 ± 4.6% and 27.1 ± 3.8%, respectively. **(c)** Alignment of K_V_2.1/Na_V_1.7 chimaera S3-S4 regions. Grey highlight indicates the residues determined by Xiao *et al*.^23^ to be key for HwTxIV functional effects on hNa_V_1.7.

The extracellular S3-S4 linker region has been demonstrated to play a key role as a binding determinant for spider venom peptides to voltage-sensing domains^18, 40^. Given that Pre1a potently affects the activation of hNa_V_1.7, we wanted to determine if this inhibition was mediated via interactions with a specific voltage-sensor domain. To this end, we used the approach of Bosmans *et al*., whereby the S3-S4 linker region of the K_V_2.1 channel was substituted with corresponding linker region of hNa_V_1.7 DI to DIV^18, 41^. The effect of Pre1a on the resultant K^+^ current was then tested for native K_V_2.1 and each of the domain chimaeras. Pre1a (1 μM) had no effect on wild-type K_V_2.1 or the DI and DIII chimaeras, however it inhibited outward current carried by the Na_V_1.7 DII and DIV/K_V_2.1 chimaeras by 44.0 ± 4.6% and 27.1 ± 3.8% (n = 6), respectively (Fig. 6a and b). Several of the residues crucial for the interaction of HwTxIV with the DII S3-S4 region of Na_V_1.7 as determine by Xiao *et al*.^23^ are also present in DIV, but not in the S3-S4 linker of Na_V_1.7 DI, DIII or Kv2.1 (Fig. 6c), which supports our observation of weaker effect of Pre1a on the DIV chimaera than the DII chimaera. Interaction with DII is completely consistent with Pre1a’s inhibition of Na_V_1.7 activation. Interestingly, the interaction with the Na_V_1.7 DIV/K_V_2.1 chimaera suggests that Pre1a should have an effect on the inactivation of hNa_V_1.7, as was noted for hNa_V_1.1 and rNa_V_1.3. However, we observed no effects of Pre1a on the inactivation of Na_V_1.7 at up to 1 μM in oocytes or 300 nM in HEK cells. These results suggest that an interaction with the S3-S4 linker alone is not sufficient to result in a potent functional effect on DIV, consistent with the effects of the spider peptide δ-Hm1a on Na_V_1.1^13^.

### Pre1a requires interactions with S3-S4 and S1-S2 regions to produce a functional effect at DIV

**Figure 7:**
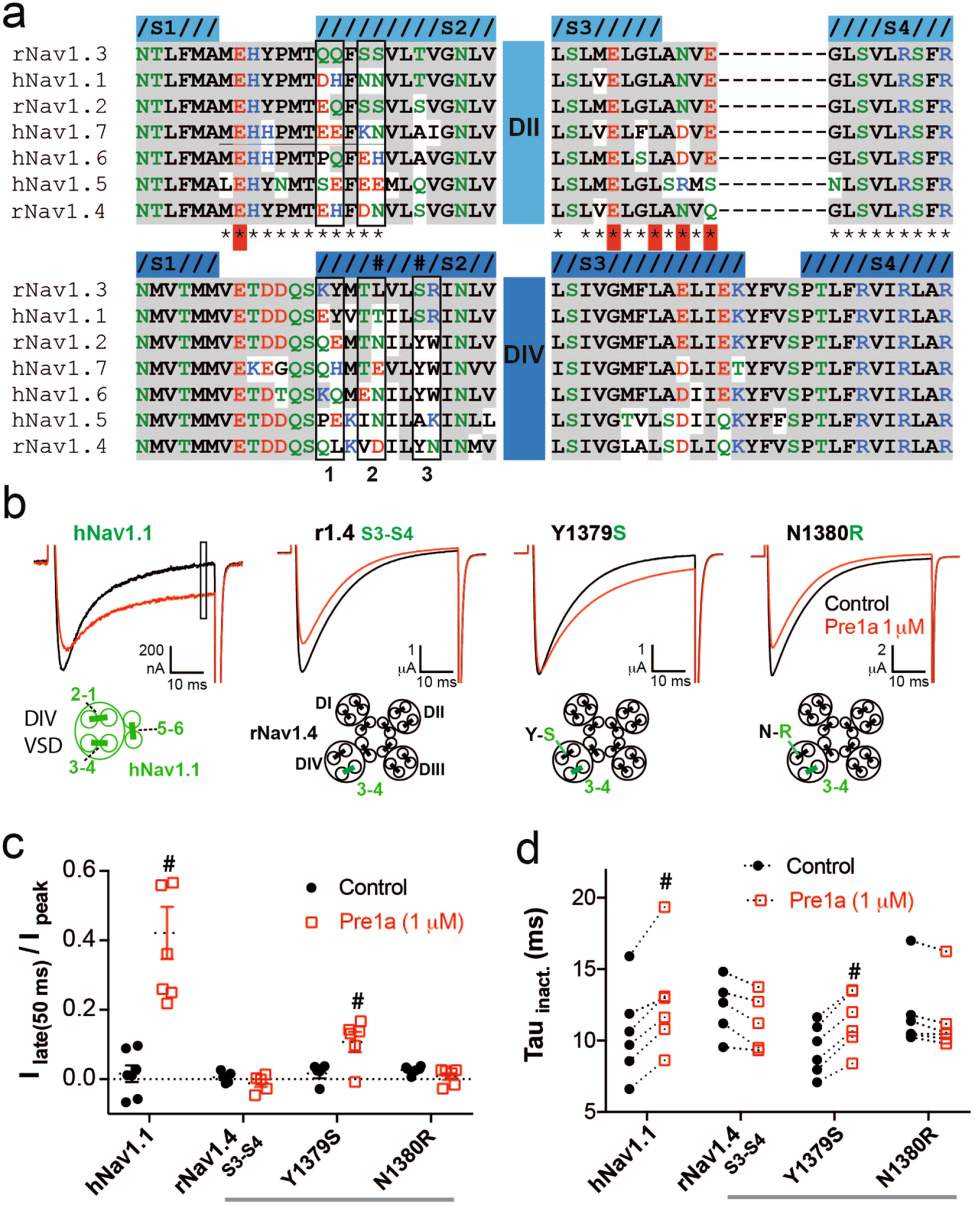
The S3-S4 linker alone accounts for the subtype selectivity of Pre1a. (**a**) Alignment of DII and DIV extracellular linkers S1-S2 and S3-S4 for Na_V_ channel isoforms used in this study. Grey shading indicates identity to rNa_V_1.3 for both domains, residue colouring indicates; **blue** = basic/positive, **red** = acidic/negative, **green** = polar, **black** = hydrophobic. Helices are defined based on the structures of rabbit Ca_V_1.1 (PDB: 5GJV) for DII, and the Na_V_Ab/hNa_V_1.7 chimaera (PDB: 5EK0) for DIV. *Indicates residues mutated in DII by Xiao *et al*.^23^, red highlight indicates importance for HwTxIV interaction. **(b**) Representative traces showing the effect of 1 µM Pre1a on hNa_V_1.1 and chimaeras of rNa_V_1.4 containing the S3-S4 linker region of Nav1.1 DIV, and additional Na_V_1.4 to 1.1 point mutants in the adjacent S1-S2 linker (schematics illustrating the chimaera constructions are shown below the respective current trace). **(c**) Normalised effect of Pre1a on the late current of channels in B (n = 5–6). **(d**) Effect of Pre1a (1 μM) on the Tau of current inactivation (determined from single exponential fit) for channels shown in 7B (n = 5–6). ^#^P < 0.05 Wilcoxon paired t-test.

The results above show that Pre1a has potent and subtype-dependent effect on Na_V_ channel gating. It potently inhibits the activation (DII effect) of hNa_V_1.1, rNa_V_1.7 and rNa_V_1.2, but not rNa_V_1.3, whereas it selectively inhibits the inactivation (DIV effect) of Na_V_1.1 and 1.3, but not 1.2 or 1.7. The striking differences in effects on Na_V_1.1, 1.2 and 1.3 are of particular significance for understanding the molecular basis of Pre1a’s subtype selectivity as these three channels have essentially identical S3-S4 linker regions in both DII and DIV (Fig. 7a) (note that Na_V_1.1 DII S3-S4 has an M to V substitution at the top of the S3 helix). Thus, this region cannot account for the substantial differences in Na_V_ channel subtype selectivity observed for Pre1a. The S1-S2 linker region has previously been shown to contribute to the interaction of HwTxIV with Na_V_1.7 via E753 a the top of S1^23^. This residue is highly conserved in all Na_V_ channels, thus it cannot account for the subtype selectivity that we observed. Figure 7a shows that there is sufficient sequence variation between Na_V_1.1, 1.2, 1.7 and 1.3 in the first half of S2 of DIV (indicated as 1, 2 and 3) to possibly explain our data. Previous mutagenesis studies on DII^23^ and DIV^13^ have shown that the divergent residues in region 1 have little effect on the activity of HwTxIV and δ-Hm1a, respectively. As region 3 of helix S2 is identical in both Na_V_1.1 and Na_V_1.3, but not 1.2 and 1.7, we assessed the potential role of these two residues (Ser/Arg) in the ability of Pre1a to inhibit inactivation. Using a chimaera-based approach we found that replacing the S3-S4 linker of rNa_V_1.4 (Pre1a insensitive) with corresponding linker from hNa_V_1.1 (Pre1a sensitive) was not sufficient to allow inhibition of inactivation by Pre1a (1 μM) (Fig. 7b,c and d). Using the rNa_V_1.4_(1.1:S3-S4)_ background, introduction of Ser1379 (S1574 in hNa_V_1.1), but not Arg1380 from Na_V_1.1 S1-S2 was sufficient for Pre1a to have a functional effect on both the extent (Fig. 7c) and rate (Fig. 7d) of fast inactivation.

### Effects of Pre1a on SH-SY5Y human neuroblastoma cells and rat dorsal root ganglion neurons

**Figure 8:**
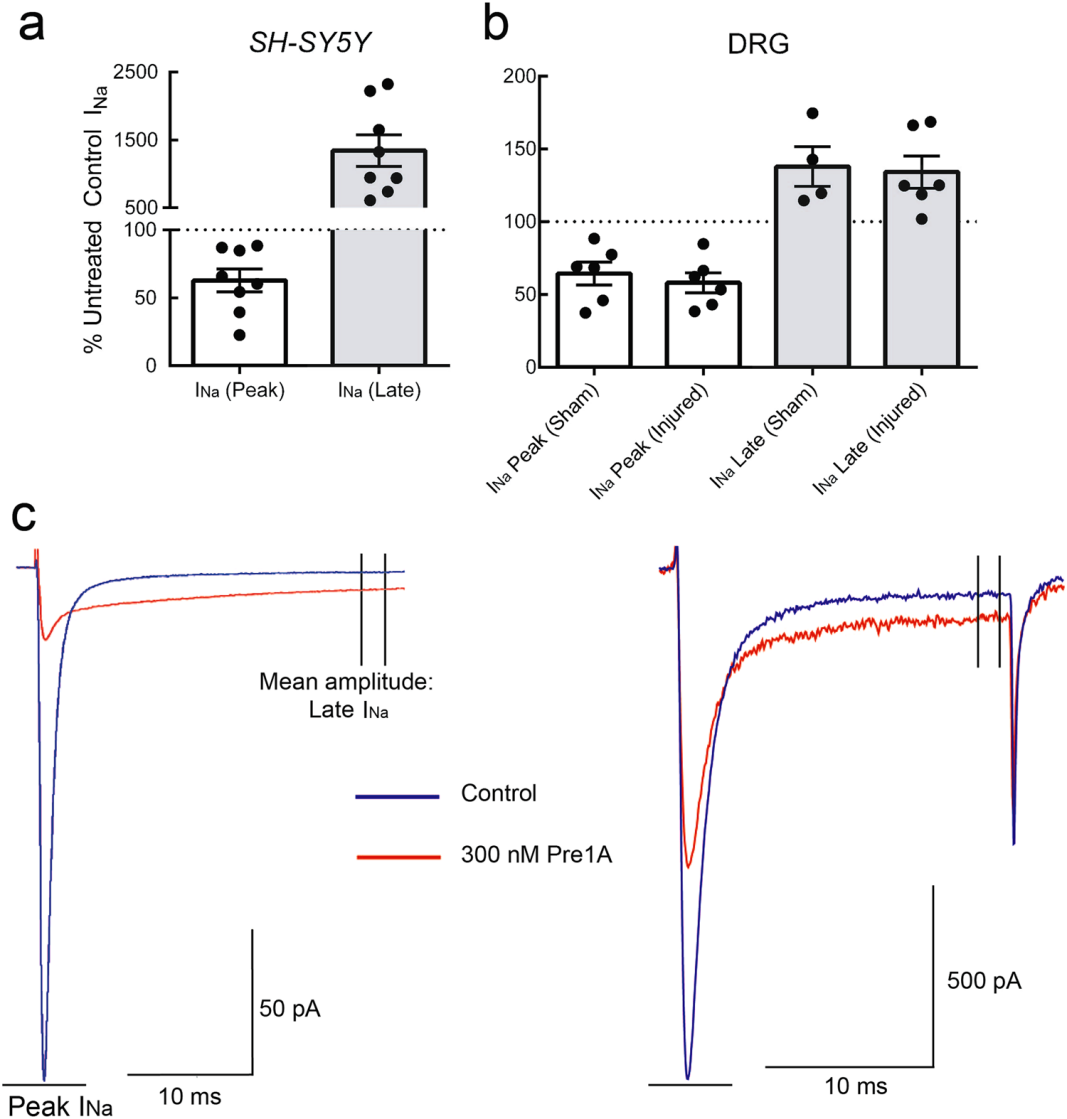
Pre1a inhibits native human and rat Na_V_ currents. **(a)** Application of 300 nM sPre1a on the human neuroblastoma cell line, *SH-SY5Y*, inhibits both peak current and fast inactivation, consistent with a Na_V_1.3 and Na_V_1.7 effect. **(b)** sPre1a (300 nM) applied to DRG neurons from sham and nerve injured rats resulted in a similar effect to that seen with *SH-SY5Y* cells. **(c)** Representative traces showing the effect of Pre1a (300 nM) on *SH-SY5Y* cell and DRG neuron from sham animal (control = blue; 300 nM sPre1a = red).

As reported previously, Na_V_1.2, Na_V_1.3 and Na_V_1.7 (but not Na_V_1.1 or Na_V_1.6) are robustly expressed in the human neuroblastoma cell line, *SH-SY5Y* ^42^. Therefore, synthetic Pre1a was tested on *SH-SY5Y* cells using manual patch clamp to assess the effects on native human Na_V_ channel currents. As shown in Fig. 8, 300 nM Pre1a inhibited the rapid peak Na^+^ current in a manner consistent with previously observed effects on rNa_V_1.2 and hNa_V_1.7, while concurrently slowing fast inactivation as was observed with rNa_V_1.3. This suggests that endogenously expressed hNa_V_1.3 exhibits similar sensitivity to Pre1a as compared to the rat isoform expressed in oocytes.

Additionally, small DRG neurons were excised from both sham and peripheral nerve ligated (PNL) adult rats, 7 days post operation. In both sets of DRG neurons, 300 nM synthetic Pre1a inhibited the peak Na^+^ current and slowed fast inactivation in a manner comparable to *SH-SY5Y* cells (Fig. 8). Na_V_1.3 expression is barely detectable in Sprague-Dawley rats at 30 days post-natal^43^ and it has been shown that this channel is upregulated and highly expressed in sensory neurons after 7–9 days post axotomy of peripheral, but not central axons^44, 45^. Therefore, minimal effects on fast inactivation of neurons from sham rats were expected, but a substantial effect on the fast inactivation of DRG neurons from post-PNL rats was expected but not observed (Fig. 8b). This result was surprising, as developmental and post-injury regulation of Na_V_1.3 would suggest this channel should not play a significant role in conductance for adult DRG neurons isolated from sham animals. We showed that Pre1a (at ~30 nM) also inhibits fast inactivation of Na_V_1.1 but not Na_V_1.6, thus the persistent current observed with Sham rat DRG neurons in the presence of Pre1a is likely due to effects on Na_V_1.1, which is present in adult neurons and has been reported to not undergo regulatory changes post-axotomy^13, 46^.

## Discussion

We have identified and characterised β/δ-TRTX-Pre1a, a tarantula venom peptide that potently modulates neuronal Na_V_ channels and possesses a unique structural and pharmacological profile. Pre1a exhibits over 70% sequence similarity with several other spider venom peptides (e.g. PaurTx3, CcoTx1, GpTx1 and Ccy1b^22, 41, 47^); however, the toxin is unique among these peptides in its ability to potently and selectively inhibit fast inactivation of Na_V_1.1 and Na_V_1.3. Furthermore, Pre1a shows a marked structural heterogeneity/flexibility in both aqueous and organic solvents, which may be related to its unique activity profile when compared to its closest sequence relatives. Thus, Pre1a represents a valuable research tool to help determine the molecular basis for spider venom peptide interactions with multiple modulatory sites in sodium channels, particularly in respect to effects on fast inactivation.

Na_V_ channel fast inactivation is controlled by the movement of the DIV voltage-sensing domain^17^. Although several spider venom peptides (such as JzTxI and JzTxII) have previously been shown to preferentially inhibit Na_V_ channel fast inactivation, they are either relatively non-selective^48^ or preferentially target Na_V_1.5^49, 50^. Two other spider peptides (ProTx-II and JzTxIV) have been found to inhibit both peak current and fast inactivation of Na_V_ channels^20, 25^, much like Pre1a. However, all these peptides share less than 50% similarity with Pre1a (See Fig. 1d) and have very different subtype selectivity profiles suggesting that Pre1a can help define the basis for subtype selective interactions with DIV. Indeed, through a series of functional experiments using channel chimaeras and point mutants, we demonstrated that a serine residue in the S2 helix of DIV that is unique to hNa_V_1.1 (S1574) and rNa_V_1.3 (S1510) is responsible for the selective modulation of fast inactivation by Pre1a. Our results are consistent with several reports suggesting that this serine residue is a major determinant for the impressively subtype selective effects of both small molecule drugs and another spider venom peptide not related to Pre1a. ICA-121431 inhibits hNa_V_1.3 and hNa_V_1.1 peak current with ~1000-fold selectivity and functions by trapping the DIV VSD in the activated state (stabilising fast inactivation, rather than inhibiting it)^51^, whereas δ-TRTX-Hm1a (Hm1a) was recently characterised as a selective inhibitor of inactivation of Na_V_1.1^13^, having been previously discovered as a low-affinity K_V_4.2 inhibitor^52^. Furthermore, a co-crystal structure of a Na_V_1.7 VSD IV chimaera with the small molecule Na_V_ channel inhibitor GX-936 has been solved revealing the detailed molecular interactions responsible for its selectivity^53^. GX-936 has the same mechanism of action as ICA-121431 thus arrests the VSD in the inactivated state as opposed to the resting state targeted by Pre1a and Hm1a. Remarkably, and despite the different state stabilised by these peptides and small molecules, they all rely on the same region in S2 (S1574 in hNa_V_1.1 and neighbouring residues) for their functional selectivity. Together, these results illustrate that the S2 helix is an important locus for subtype selective modulation of Na_V_ channel fast inactivation and that the key residues involved appear to be accessible to ligands in both the resting and inactivated states.

Although Hm1a and Pre1a share the ability to inhibit Na_V_1.1 fast inactivation, they differ greatly in primary sequence (only 17% similarity when excluding the structurally critical Cys residues). Interestingly, the small amount of similarity that does exist between them is a hydrophobic patch clustered in loop 1 of the peptide (Fig. 1d). Co-incidentally this is the region of Pre1a that is structurally mobile, a characteristic that has not been reported for venom peptides most closely related to Pre1a and that seem to predominantly inhibit channel activation^22, 41, 47^. From extensive structure-activity studies carried out by several pharmaceutical companies on spider venom peptides closely related to Pre1a (i.e. GpTx1, CcoTx1 and HwTxIV^26–28, 34^), the aromatic residues at the tip of Loop 1 are key residues for the interaction of these peptides with Na_V_ channels, in particular for their inhibitory effects on channel activation. Whether the flexibility of loop 1 in Pre1a has any role in its ability to potently interact with both DII and/or DIV VSDs (i.e., resulting in two conformations of the same pharmacophore residues) remains to be elucidated.

The interactions of spider venom peptides that inhibit Na_V_ channel activation have been the focus of extensive chimaera and scanning mutagenesis studies to characterise the binding site on the channel. Bosmans *et al*. demonstrated that the S3-S4 linker region of any of DI to DIII can be a primary binding determinant for a number of venom peptides by transplanting this region in to a toxin insensitive background and transferring functional peptide binding^18^. Subsequently, a motif, which spans the DII S1-S2 *and* S3-S4 regions, that is critical for the inhibitory activity of HwTx-IV on hNa_V_1.7 was identified and consists of the residues E753 in S1, and E811, L814, D816, E818 in S3-S4^23^ (See red asterisks in Fig. 7a). This has since been functionally demonstrated for two other spider peptides with substantial similarity to Pre1a^54, 55^ as well as suggested structurally (in solution using NMR) for the interaction of VSTX1 and the VSD of K_V_Ap^32^. Our data showing a greater effect of Pre1a on the DII Kv2.1/hNav1.7 S3-S4 chimaera than that containing the DIV S3-S4 linker is in accord with the these previous studies. However, interaction with the residues identified in S1 and S3-S4 does not explain the substantial differences in Pre1a’s ability to inhibit Na_V_ channel activation and despite the studies carried out to date, little insight into the basis of Na_V_ channel subtype selectivity has been gained in regards to DII. Pre1a most potently inhibited the activation of Na_V_1.7 and Na_V_1.2 with ~70-fold selectivity over Na_V_1.3. The latter two channels have identical sequences in the S3-S4 linker, thus binding to this region cannot account for the observed selectivity. Interestingly hNa_V_1.1 and rNa_V_1.2 only differ by only two residues in the S2 helix, a conservative Thr (1.3) to Ser (1.2) substitution (at the position corresponding to the Ser1574 in Nav1.1 DIV) and a less conservative Gln (1.3) to Glu (1.2) substitution at the top of S2. This strongly suggests that residues in one or both of these positions make major contributions to the interaction of Pre1a with Na_V_ VSD (driven primarily by interactions with S3-S4) that together result in functional inhibition of the channel. Thus, similar to what we showed for the molecular basis of sub-type selective inhibition of inactivation, subtype selective interactions at DII are also likely determined by the variability in the S2 helix.

In conclusion, we have identified a novel spider venom peptide and demonstrated how its so far unique activity on the Na_V_ channel family can be used to gain insight into the molecular basis of subtype selectivity. It should be noted that few venom peptide modulators of Na_V_ channels have been studied in the same detail as reported here, thus the structural heterogeneity and binding site promiscuity of Pre1a may indeed be a more common property than we realise. Pre1a has great value as a research tool for exploration of the selectivity profile of activation and inactivation on different Na_V_ channel isoforms, as well as exploring the consequences and effects of conformational flexibility to activity in this family of channel modulators.

## Methods

### Venom peptide purification

*Psalmopoeus reduncus* venom was purchased from SpiderPharm (Yarnell). Venom was fractionated using a C18 218TP54 column (4.6 × 250 mm, 5 μm, Grace Discovery Sciences) with solvent A (H_2_O, 0.05% TFA) and B (90% acetonitrile, 0.045% TFA) over a gradient of solvents (15–40% B in 36 min, 40–100% B in 12.5 min at 1 ml/min). Fractions were collected, dried and assayed for activity against hNa_V_1.7 expressed in *Xenopus laevis* oocytes. The active fractions were further separated on a PromixMP column (4.6 × 250 mm, Sielc) with a gradient of 10–35% B in 35 min at 1 ml/min, then a Prosphere C4 column (3.0 × 150 mm, Grace Discovery Sciences) with a gradient of 20–45% B at 0.75 ml/min.

Peptides were analysed using MALDI-TOF mass spectrometry (Applied Biosystems 4700 Proteomics Bioanalyser) in reflector mode using α-cyano-4-hydroxy-cinnamic acid (CHCA, 5 mg/ml in 60:40 solvent B:A). Reduction/alkylation and sequencing of approximately 3 μg of the peptide was carried out by N-terminal Edman degradation.

### Peptide synthesis

Synthetic Pre1a was assembled manually using Boc SPPS chemistry as described previously^56^. The side-chain protecting groups chosen were Asn (Xan), Arg (Tos), Asp (OcHex), Cys (4-MeBzl), Lys (ClZ), Ser (Bzl), Trp (CHO) and Tyr (BrZ). The crude reduced peptide was purified using preparative reversed-phase chromatography (Vydac C18 218TP1022), using a gradient of 0–80% B over 80 min). The peptide was oxidised at a concentration of 0.02 mM in either aqueous 0.33 M NH_4_OAc/0.5 M GnHCI or 2 M NH_4_OH/0.1 M NH_4_OAc at pH 7.8, 4 °C in the presence of both reduced and oxidised glutathione (peptide:GSH:GSSG, 1:100:10, molar ratio). Oxidised peptides were purified using preparative RP-HPLC.

### Bacterial recombinant production of Pre1a

Pre1a was produced recombinantly using an *E. coli* periplasmic expression system as described previously^57^. Briefly, a synthetic gene encoding Pre1a was codon optimised for bacterial expression and subcloned into a pLicC-His_6_-MBP periplasmic expression vector, where the peptide is expressed as a C-terminal fusion to His_6_-tagged maltose binding protein (MBP) separated by a tobacco etch virus (TEV) protease cleavage site, leaving an additional N-terminal *Ser* after cleavage. Fusion proteins were expressed in *E. coli* strain BL21(λDE3) and isolated from cell lysates using Ni-NTA Superflow resin (Qiagen). The His_6_-MBP tag was removed from the fusion protein using TEV protease, and recombinant Pre1a purified using RP-HPLC.

For production of uniformly ^13^C/^15^N-labelled Pre1a, cultures were grown in minimal medium supplemented with ^13^C_6_-glucose and ^15^NH_4_Cl as the sole carbon and nitrogen sources, respectively. In order to facilitate comparisons between synthetic and recombinant Pre1a, residue numbers for the native toxin are used throughout the text even though the recombinant toxin contains an additional N-terminal serine residue that is a vestige of the TEV cleavage site.

### Two-electrode voltage clamp analysis on Na_V_ channel-expressing oocytes

The preparation and injection of *Xenopus laevis* oocytes and Na_V_ channel recordings using two-electrode voltage-clamp (TEVC) were carried out as described previously^58^. Capped cRNA encoding rat Na_V_1.2, Na_V_1.3 and Na_V_1.4, and human Na_V_1.5 and Na_V_1.7 were injected at 20–40 ng of cRNA/oocyte and kept at 17–18 °C in ND96 solution containing (in mM) 96 NaCl, 2 KCl, 1 CaCl_2_, 2 MgCl_2_, 5 HEPES, 5 pyruvic acid, 50 µg/ml gentamicin (pH 7.4), and horse serum (2.5%). Membrane currents were recorded 2–5 days after injection. Oocytes were clamped at −90 mV and currents were elicited by a 50 ms depolarising step to −10 or 0 mV every 10 s for concentration response curves. Both peak and late current (50 ms post peak) were analysed. The concentration response curves were fitted to the Hill equation (GraphPad Prism 6). Current-voltage (*I-V*) relationships and voltage-dependence of fast inactivation were determined using a family of 100 ms conditioning pulses from −100 mV to +50 mV (+60 mV for rNa_V_1.3) in 5 mV steps, followed by a test depolarisation step to 0 mV with a 20 s sweep interval from a holding potential of −90 mV. To determine the voltage-dependence of activation non-normalised peak current was used from the *I-V* family to calculate channel conductance using the equation: *G*(*V*) = *I*/(*V* − *V*_*rev*_), in which *I*, *V*, and *V*_*rev*_ represent inward Na_V_ current, test potential, and reversal potential, respectively. The half-activation potential (*V*_*1/2*_) was determined using a Boltzmann fit (GraphPad Prism 6). All data points are shown as the pIC_50_ ± S.E.M., as well as the IC_50_. Replicates (*n*) represent separate experimental oocytes. Pre1a was resuspended in ND96 solution containing 0.1% bovine serum albumin for all activity analysis. Construction and recording of the K_V_2.1chimaeras containing hNa_V_1.7 DI-DIV S3-S4 linker regions was completed as described previously^18, 41^. Design and construction of the hNa_V_1.1/rNa_V_1.4 DIV chimaeras is detailed in Osteen *et al*.^13^. Not all isoforms of Na_V_s from the same organism were available at the time of experimentation. Even though different species (rat and human) were used, careful analysis of the data in relation to the sequence differences of the isoforms used, still yields valuable insights in to the molecular basis of peptide:channel interaction.

### NMR data acquisition and structural analysis

^13^C/^15^N-labelled peptide in 20 mM potassium phosphate buffer, pH 5 containing 5% D_2_O was filtered using a low-protein-binding Ultrafree-MC centrifugal filter (0.22 µm pore size; Millipore, MA, USA), then 300 μL of ~300 μM was added to a susceptibility-matched 5 mm outer-diameter microtube (Shigemi Inc.). NMR spectra were acquired at 298 K on a 900 MHz Bruker AVANCE II+ spectrometer (Bruker BioSpin) equipped with a cryogenically cooled probe. Data used for resonance assignment were acquired using non-uniform sampling (NUS); sampling schedules that approximated the rate of signal decay along the various indirect dimensions were generated using sched3D^59^. The decay rates used were 1 Hz for all constant-time ^15^N dimensions, 30 Hz for all ^13^C dimensions, and 15 Hz for the semi-constant indirect ^1^H dimension of the H(CC) (CO)NH/(H)CC(CO)NH-TOCSY experiments. ^13^C- and ^15^N-edited HSQC-NOESY experiments were acquired using linear sampling. Separate experiments were acquired for the aliphatic and aromatic regions of the ^13^C dimension.

NUS data were processed using the Rowland NMR toolkit (www.rowland.org/rnmrtk/toolkit.html); maximum entropy parameters were selected automatically as described^60^. NMR spectra were analysed and assigned using the program XEASY^61^ or SPARKY^62^. ^1^H_N_, ^15^N, ^13^C_α_, ^13^C_β_, and ^13^C′ resonance assignments were obtained from analysis of amide-proton strips in 3D HNCACB, CBCA(CO)NH, and HNCO spectra. Side-chain ^1^H and ^13^C chemical shifts were obtained primarily from 3D H(CC) (CO)NH-TOCSY and (H)CC(CO)NH-TOCSY spectra, respectively. The remaining side-chain assignments were derived from 3D ^15^N- and ^13^C-edited NOESY-HSQC spectra. The program TALOS+ ^31^ was used to predict the secondary structure of the peptide. Disulfide connectives were determined from NOESY patterns^59^. During the automated NOESY assignment/structure calculation process, CYANA assigned 90.1% of all NOESY cross-peaks (829 of 920).

### Automated patch clamp analyses on Na_V_ channel-expressing mammalian cells

For patch clamp analysis, HEK293 cells stably co-expressing the hNa_V_1.1, hNa_V_1.6, or hNa_V_1.7 α-subunit with the Na_V_β_1_-subunit (SB Drug Discovery) were cultured following manufacturer guidelines. Cells were removed from culture at 70% confluency using Detachin (Genlantis) and resuspended to 5 × 10^6^ cells/mL in Ex-Cell ACF CHO Medium (Life Technologies) supplemented with 25 mM HEPES (Sigma-Aldrich) and 1 × Glutamax (Life Technologies) before being transferred to the Q-Patch *Q*-*Stirrer* and allowed to recover for 30 min before assay.

The external solution contained (in mM): 140 NaCl, 4 KCl, 2 CaCl_2_, 1 MgCl_2_, 10 HEPES, 20 TEA-Cl, 10 glucose, pH 7.4 (with NaOH) and adjusted to 315 mOsm 0.05% BSA was added to prevent adsorptive loss of peptide. The intracellular solution contained (in mM): 140 CsF, 1/5 EGTA/CsOH, 10 HEPES and 10 NaCl, pH 7.4 (with CsOH) and adjusted to 320 mOsm. Whole-cell patch-clamp experiments were performed at room temperature on a QPatch-16 automated electrophysiology platform (Biolin Scientific) using 16-channel planar patch chip plates (*Q-Plates*) with a patch hole diameter of 1 µm and resistance of 2 ± 0.1 MΩ. Cell positioning and sealing parameters were set as follows: positioning pressure −60 mbar, minimum seal resistance 0.1 GΩ, holding potential −90 mV and holding pressure −20 mbar. Whole-cell currents were filtered at 5 kHz (8-pole Bessel) and digitised at 25 kHz. A *P/6* online leak-subtraction protocol was used with non-leak-subtracted currents acquired in parallel. Cells were maintained with holding potentials of −80 (hNa_V_1.1) or −100 mV (hNa_V_1.6 and hNa_V_1.7) between protocols, respectively and depolarised to −10 mV with a sweep interval of 20 s to allow complete recovery from inactivation between sweeps. Pre1a concentrations were incubated for a fixed time of 5–10 min to allow steady-state activity, monitored by the *I-T* plot.

### Patch clamp analyses of SH-SY5Y cells and rat DRG neurons

Whole-cell patch clamp electrophysiology was carried out on *SH-SY5Y* cells as described previously^42^ or from small (<25 μm diameter) acutely isolated adult male rat DRG neurons as described previously^63^. DRG neurons were taken from rats that had undergone either partial ligation of the left sciatic nerve (PNL) to induce a state of neuropathic pain (defined as development of significant mechanical allodynia seven days after surgery) or sham operated rats^64^. *SH-SY5Y* cells were used within 24–72 h, and DRG neurons were used within 6 hours of plating. Only cells with minimal or no processes were used. Whole-cell patch-clamp recordings were performed at room temperature with fire-polished patch electrodes prepared from borosilicate glass (SDR Clinical Technology). Electrode resistance was 3.5–5 MΩ when filled with an internal solution containing (composition in mM): 120 CsCl, 5 MgATP, 5 NaCl, 2 CaCl_2_, 20 HEPES, 10 EGTA, pH 7.3 and adjusted to 283–286 mOsm. Cells were continuously bathed in a HEPES-buffered physiological saline (HBS; composition in mM): 155 NaCl, 2.5 KCl, 1.8 CaCl_2_, 1.2 MgCl_2_, 10 HEPES, 10 glucose, pH 7.4 (adjusted with NaOH) and adjusted to 328–331 mOsm. Currents were recorded using a HEKA EPC-9 amplifier and PULSE software (v8.8, HEKA Elektronik, Lambrecht/Pfalz, Germany). Data was filtered at 4 kHz and sampled at 20 kHz. Series resistance was compensated by 80%. Capacitance transients were compensated and leak subtraction was performed with a *P/8* online protocol. Drugs were applied to cells using a gravity-fed superfusion system (250 μm diameter) positioned directly above the cell resulting in rapid solution exchange (<500 ms).

### Ethics statement

All animal experiments complied with the Australian code of practice for the care and use of animals for scientific purposes, (8^th^ Ed. 2013) and great care was taken to minimise animal suffering at all times. Experiments involving rats were approved by the University of Sydney (Approval number K00/1-2009/3/4940) or Royal North Shore Hospital/University of Technology Animal Ethics Committee (Approval number 0411-067A). Experiments using *X. laevis* were approved by The University of Queensland Animal Ethics Committee (Approval Number: QBI/059/13/ARC/NHMRC). Oocytes were obtained via recovery surgery performed under tricaine methanesulfonate (MS-222) anaesthesia (animals bathed in 1.3 mg/ml solution). Minimum time between surgeries on the same animal was six months.

## Electronic supplementary material

Supplementary data

## References

[1] Catterall, W. A., Goldin, A. L. & Waxman, S. G. *Voltage-gated sodium channels, introduction*, http://www.guidetopharmacology.org/GRAC/FamilyIntroductionForward?familyId=82. (2016).

[2] Vetter, I. *et al*. Na_V_1.7 as a pain target - from gene to pharmacology. *Pharmacol. Ther*. In press, doi:10.1016/j.pharmthera.2016.11.015 (2016). 10.1016/j.pharmthera.2016.11.015 27916648

[3] Gilchrist J (2014). Na_V_1.1 modulation by a novel triazole compound attenuates epileptic seizures in rodents. ACS Chem. Biol..

[4] Wingerd, J. S., Vetter, I. & Lewis, R. J. In *Therapeutic Targets* 63–122 (John Wiley & Sons, Inc., 2012).

[5] Dib-Hajj SD, Yang Y, Black JA, Waxman SG (2012). The Na_V_1.7 sodium channel: from molecule to man. Nature Reviews Neuroscience.

[6] Yang Y (2004). Mutations in SCN9A, encoding a sodium channel alpha subunit, in patients with primary erythermalgia. J Med Genet.

[7] Fertleman CR (2006). SCN9A mutations in paroxysmal extreme pain disorder: allelic variants underlie distinct channel defects and phenotypes. Neuron.

[8] Cox JJ (2006). An SCN9A channelopathy causes congenital inability to experience pain. Nature.

[9] Amaya F (2006). The voltage-gated sodium channel Na_V_1.9 is an effector of peripheral inflammatory pain hypersensitivity. J. Neurosci..

[10] Hains BC, Saab CY, Waxman SG (2005). Changes in electrophysiological properties and sodium channel Na_V_1.3 expression in thalamic neurons after spinal cord injury. Brain.

[11] Kim CH, Oh Y, Chung JM, Chung K (2001). The changes in expression of three subtypes of TTX sensitive sodium channels in sensory neurons after spinal nerve ligation. Brain Res. Mol. Brain Res..

[12] Leo S, D’Hooge R, Meert T (2010). Exploring the role of nociceptor-specific sodium channels in pain transmission using Nav1.8 and Nav1.9 knockout mice. Behav. Brain. Res..

[13] Osteen JD (2016). Selective spider toxins reveal a role for the Nav1.1 channel in mechanical pain. Nature.

[14] Vetter I (2011). Venomics: a new paradigm for natural products-based drug discovery. Amino Acids.

[15] Saez NJ (2010). Spider-Venom Peptides as Therapeutics. Toxins.

[16] Das, S., Gilchrist, J., Bosmans, F. & Van Petegem, F. Binary architecture of the Na_v_1.2-beta2 signaling complex. *Elife* **5**, doi:10.7554/eLife.10960 (2016). 10.7554/eLife.10960 PMC4769172 26894959

[17] Ahern CA, Payandeh J, Bosmans F, Chanda B (2016). The hitchhiker’s guide to the voltage-gated sodium channel galaxy. The Journal of General Physiology.

[18] Bosmans F, Martin-Eauclaire MF, Swartz KJ (2008). Deconstructing voltage sensor function and pharmacology in sodium channels. Nature.

[19] Capes DL, Goldschen-Ohm MP, Arcisio-Miranda M, Bezanilla F, Chanda B (2013). Domain IV voltage-sensor movement is both sufficient and rate limiting for fast inactivation in sodium channels. The Journal of General Physiology.

[20] Wang M (2008). JZTX-IV, a unique acidic sodium channel toxin isolated from the spider Chilobrachys jingzhao. Toxicon.

[21] Bosmans F, Swartz KJ (2010). Targeting voltage sensors in sodium channels with spider toxins. Trends in Pharmacological Sciences.

[22] Bosmans F (2006). Four novel tarantula toxins as selective modulators of voltage-gated sodium channel subtypes. Mol Pharmacol.

[23] Xiao Y, Jackson JO, Liang S, Cummins TR (2011). Common molecular determinants of tarantula Huwentoxin-IV inhibition of Na^+^ channel voltage sensors in domains II and IV. Journal of Biological Chemistry.

[24] Corzo G, Escoubas P (2003). Pharmacologically active spider peptide toxins. CMLS, Cell. Mol. Life Sci..

[25] Xiao Y, Blumenthal K, Jackson JO, Liang S, Cummins TR (2010). The Tarantula Toxins ProTx-II and Huwentoxin-IV Differentially Interact with Human Na_v_1.7 Voltage Sensors to Inhibit Channel Activation and Inactivation. Molecular Pharmacology.

[26] Murray JK (2016). Single Residue Substitutions That Confer Voltage-Gated Sodium Ion Channel Subtype Selectivity in the Na_V_1.7 Inhibitory Peptide GpTx-1. J. Med. Chem..

[27] Revell JD (2013). Potency optimization of Huwentoxin-IV on hNav1.7: A neurotoxin TTX-S sodium-channel antagonist from the venom of the Chinese bird-eating spider Selenocosmia huwena. Peptides.

[28] Shcherbatko A (2016). Engineering Highly Potent and Selective Microproteins Against Nav1.7 Sodium Channel for Treatment of Pain. Journal of Biological Chemistry.

[29] King GF, Gentz MC, Escoubas P, Nicholson GM (2008). A rational nomenclature for naming peptide toxins from spiders and other venomous animals. Toxicon.

[30] Güntert, P. In *Protein NMR Techniques* Vol. 278 *Methods in Molecular Biology™* (ed. A. Kristina Downing) Ch. 17, 353–378 (Humana Press, 2004).

[31] Shen Y, Delaglio F, Cornilescu G, Bax A (2009). TALOS+: a hybrid method for predicting protein backbone torsion angles from NMR chemical shifts. Journal of biomolecular NMR.

[32] Lau CH, King GF, Mobli M (2016). Molecular basis of the interaction between gating modifier spider toxins and the voltage sensor of voltage-gated ion channels. Sci. Rep.

[33] Klint JK, Chin YK, Mobli M (2015). Rational Engineering Defines a Molecular Switch That Is Essential for Activity of Spider-Venom Peptides against the Analgesics Target NaV1.7. Mol. Pharmacol..

[34] Minassian NA (2013). Analysis of the Structural and Molecular Basis of Voltage-sensitive Sodium Channel Inhibition by the Spider Toxin Huwentoxin-IV (μ-TRTX-Hh2a). Journal of Biological Chemistry.

[35] Arnold K, Bordoli L, Kopp J, Schwede T (2006). The SWISS-MODEL workspace: a web-based environment for protein structure homology modelling. Bioinformatics.

[36] Gilchrist J, Das S, Van Petegem F, Bosmans F (2013). Crystallographic insights into sodium-channel modulation by the beta4 subunit. Proc. Natl. Acad. Sci. USA.

[37] He B, Soderlund DM (2011). Differential state-dependent modification of rat Nav1.6 sodium channels expressed in human embryonic kidney (HEK293) cells by the pyrethroid insecticides tefluthrin and deltamethrin. Toxicology and Applied Pharmacology.

[38] Goldin, A. L. In *Expression and Analysis of Recombinant Ion Channels: From Structural Studies to Pharmacological Screening* (eds Derek J. Trezise, Jeffrey J. Clare) 1–25 (Wiley-VCH Verlag, Weinheim, 2006).

[39] Witchel HJ, Milnes JT, Mitcheson JS, Hancox JC (2002). Troubleshooting problems with *in vitro* screening of drugs for QT interval prolongation using HERG K+ channels expressed in mammalian cell lines and Xenopus oocytes. Journal of Pharmacological and Toxicological Methods.

[40] Li-Smerin Y, Swartz KJ (2000). Localization and molecular determinants of the Hanatoxin receptors on the voltage-sensing domains of a K(+) channel. J. Gen. Physiol..

[41] Klint JK (2015). Seven novel modulators of the analgesic target NaV1.7 uncovered using a high-throughput venom-based discovery approach. British Journal of Pharmacology.

[42] Vetter I (2012). Characterisation of Nav types endogenously expressed in human SH-SY5Y neuroblastoma cells. Biochemical Pharmacology.

[43] Felts PA, Yokoyama S, Dib-Hajj S, Black JA, Waxman SG (1997). Sodium channel alpha-subunit mRNAs I, II, III, NaG, Na6 and hNE (PN1): different expression patterns in developing rat nervous system. Brain Res Mol Brain Res.

[44] Waxman SG, Kocsis JD, Black JA (1994). Type III sodium channel mRNA is expressed in embryonic but not adult spinal sensory neurons, and is reexpressed following axotomy. J Neurophysiol.

[45] Black JA (1999). Upregulation of a Silent Sodium Channel After Peripheral, but not Central, Nerve Injury in DRG Neurons. Journal of Neurophysiology.

[46] Wang W, Gu J, Li Y-Q, Tao Y-X (2011). Are voltage-gated sodium channels on the dorsal root ganglion involved in the development of neuropathic pain?. Molecular Pain.

[47] Murray JK (2015). Engineering Potent and Selective Analogues of GpTx-1, a Tarantula Venom Peptide Antagonist of the NaV1.7 Sodium Channel. Journal of Medicinal Chemistry.

[48] Tao H (2016). Molecular determinant for the tarantula toxin Jingzhaotoxin-I slowing the fast inactivation of voltage-gated sodium channels. Toxicon.

[49] Wang M (2008). Jingzhaotoxin-II, a novel tarantula toxin preferentially targets rat cardiac sodium channel. Biochemical Pharmacology.

[50] Tang C (2014). The tarantula toxin jingzhaotoxin-XI (κ-theraphotoxin-Cj1a) regulates the activation and inactivation of the voltage-gated sodium channel Nav1.5. Toxicon.

[51] McCormack K (2013). Voltage sensor interaction site for selective small molecule inhibitors of voltage-gated sodium channels. Proceedings of the National Academy of Sciences.

[52] Escoubas P, Diochot S, Célérier M-L, Nakajima T, Lazdunski M (2002). Novel Tarantula Toxins for Subtypes of Voltage-Dependent Potassium Channels in the Kv2 and Kv4 Subfamilies. Molecular Pharmacology.

[53] Ahuja, S. *et al*. Structural basis of Nav1.7 inhibition by an isoform-selective small-molecule antagonist. *Science* **350**, aac5464, doi:10.1126/science.aac5464 (2015). 10.1126/science.aac5464 26680203

[54] Cai T (2015). Mapping the interaction site for the tarantula toxin hainantoxin-IV (β-TRTX-Hn2a) in the voltage sensor module of domain II of voltage-gated sodium channels. Peptides.

[55] Liu Z (2013). Structure and Function of Hainantoxin-III, a Selective Antagonist of Neuronal Tetrodotoxin-sensitive Voltage-gated Sodium Channels Isolated from the Chinese Bird Spider Ornithoctonus hainana. Journal of Biological Chemistry.

[56] Klint JK (2014). Isolation, synthesis and characterization of ω-TRTX-Cc1a, a novel tarantula venom peptide that selectively targets L-type CaV channels. Biochemical Pharmacology.

[57] Klint JK (2013). Production of Recombinant Disulfide-Rich Venom Peptides for Structural and Functional Analysis via Expression in the Periplasm of *E. coli*. PLoS One.

[58] Chow C, Cristofori-Armstrong B, Undheim E, King G, Rash L (2015). Three Peptide Modulators of the Human Voltage-Gated Sodium Channel 1.7, an Important Analgesic Target, from the Venom of an Australian Tarantula. Toxins.

[59] Mobli M, Stern AS, Bermel W, King GF, Hoch JC (2010). A non-uniformly sampled 4D HCC(CO)NH-TOCSY experiment processed using maximum entropy for rapid protein sidechain assignment. Journal of Magnetic Resonance.

[60] Mobli M, Maciejewski MW, Gryk MR, Hoch JC (2007). An automated tool for maximum entropy reconstruction of biomolecular NMR spectra. Nat Methods.

[61] Bartels C, Xia TH, Billeter M, Guntert P, Wuthrich K (1995). The program XEASY for computer-supported NMR spectral analysis of biological macromolecules. Journal of biomolecular NMR.

[62] Goddard, T. D. & Kneller, D. G. *SPARKY 3, NMR Assignment and Integration Software* (University of California, San Francisco) https://www.cgl.ucsf.edu/home/sparky/ (2008).

[63] Murali SS, Napier IA, Rycroft BK, Christie MJ (2012). Opioid-related (ORL1) receptors are enriched in a subpopulation of sensory neurons and prolonged activation produces no functional loss of surface N-type calcium channels. The Journal of Physiology.

[64] Seltzer Ze, Dubner R, Shir Y (1990). A novel behavioral model of neuropathic pain disorders produced in rats by partial sciatic nerve injury. Pain.

[65] Meir, A., Cherki, R. S., Kolb, E., Langut, Y. & Bajayo, N. Novel peptides isolated from spider venom, and uses thereof. (2011).

[66] Middleton RE (2002). Two tarantula peptides inhibit activation of multiple sodium channels. Biochemistry.

[67] Peng K, Shu Q, Liu Z, Liang S (2002). Function and Solution Structure of Huwentoxin-IV, a Potent Neuronal Tetrodotoxin (TTX)-sensitive Sodium Channel Antagonist from Chinese Bird Spider Selenocosmia huwena. Journal of Biological Chemistry.

[68] Xiao Y-C, Liang S-P (2003). Purification and characterization of Hainantoxin-V, a tetrodotoxin-sensitive sodium channel inhibitor from the venom of the spider Selenocosmia hainana. Toxicon.

