# Supplementary material for: The tarantula toxin β/δ-TRTX-Pre1a highlights the importance of the S1-S2 voltage-sensor region for sodium channel subtype selectivity
Source: Sci Rep. 2017 Apr 20;7:974. doi: 10.1038/s41598-017-01129-0 (PMC5430537; doi:10.1038/s41598-017-01129-0)
Supplement: Supplementary file 1 — Supplementary data [file 41598_2017_1129_MOESM1_ESM.pdf]

Supplementary data for

The tarantula toxin *β/δ-TRTX-Pre1a* highlights the importance of the S1-S2 voltage-sensor region for sodium channel subtype selectivity.

Joshua S. Wingerd[1], Christine A. Mozar[4], Christine A. Üssing[1], Swetha S. Murali[4], Yanni K.-Y. Chin[1], Ben Cristofori-Armstrong[1], Thomas Durek[1], John Gilchrist[7], Christopher W. Vaughan[5], Frank Bosmans[7], David J. Adams[6], Richard J. Lewis[1], Paul F. Alewood[1], Mehdi Mobli[2], Macdonald J. Christie[4], Lachlan D. Rash[3,1]*

[1] Institute for Molecular Bioscience; [2] Centre for Advanced Imaging & School of Chemistry and Molecular Biosciences; [3] School of Biomedical Sciences, The University of Queensland, Brisbane, Queensland 4072, Australia

[4] Discipline of Pharmacology and [5] Pain Management Research Institute, University of Sydney, New South Wales 2006, Australia

[6] Illawarra Health and Medical Research Institute, University of Wollongong, Wollongong NSW 2522, Australia

[7] Department of Physiology and Solomon H. Snyder Department of Neuroscience, Johns Hopkins University School of Medicine, Baltimore, Maryland 21205, USA.

*Correspondence to: Lachlan D. Rash, Tel +61 7 3346 2985; Email l.rash@uq.edu.au

Current addresses:
SSM: Harvard Medical School, Children's Hospital, 300 Longwood Ave, Boston MA 02115.
CAU: Novo Nordisk A/S, Copenhagen Area, Capital Region, Denmark.

Key Words: TRTX-Pre1a, tarantula, spider, venom, toxin, peptide, voltage-gated sodium channels

A

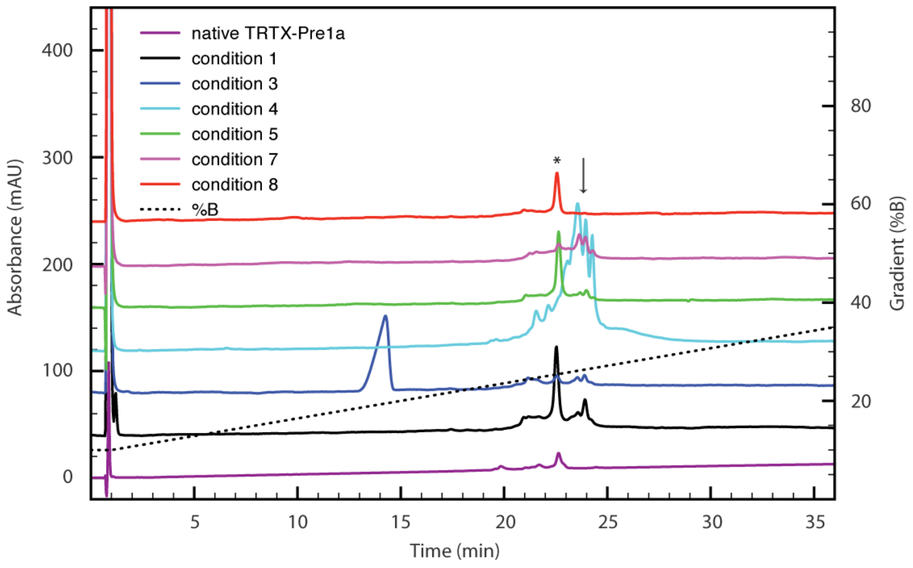

B

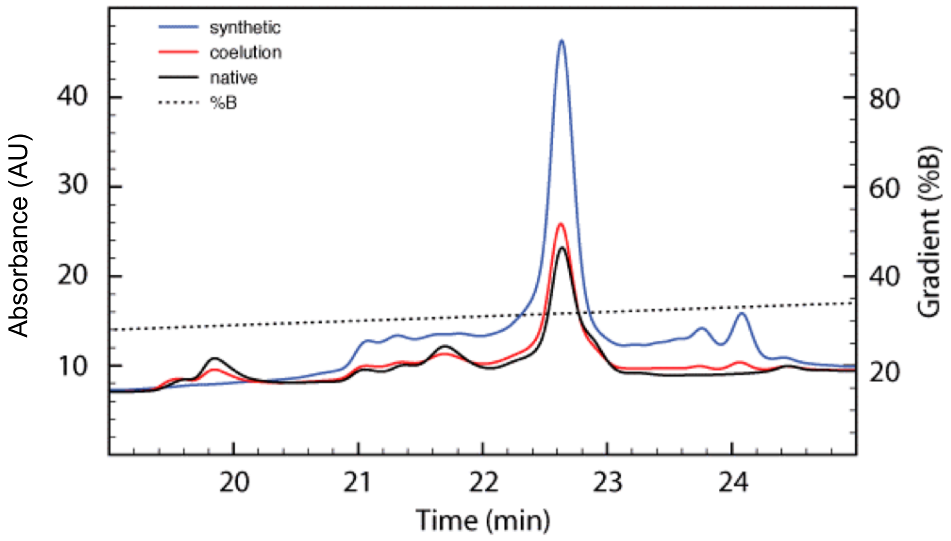

Supplementary Figure 1. A) Analysis of oxidation conditions by RP-HPLC after 10 days of oxidation at 4°C. Samples were prepared by adding 1 µL TFA to 19 µL aliquots of oxidation mixtures. Samples were analysed on a Zorbax C18 column (4.6 × 50 mm, 5 µm). Applying an injection volume of 15 µL, flow rate 1 mL/min and a linear gradient of 1%/min (15-45 %B) depicted by dotted line. Fully reduced TRTX-Pre1a has a retention time of 24 min (indicated by arrow), while major isomer of oxidised Pre1a displayed a left-shift in retention time to approximately 22.5 min (indicated by asterisk). Native TRTX-Pre1a displays the same retention time as major isomer in oxidation of synthetic TRTX-Pre1a. **B)** Co-injection of synthetic Pre1a (blue) with peptide isolated from the venom (black) yields co-elution of peaks (red).

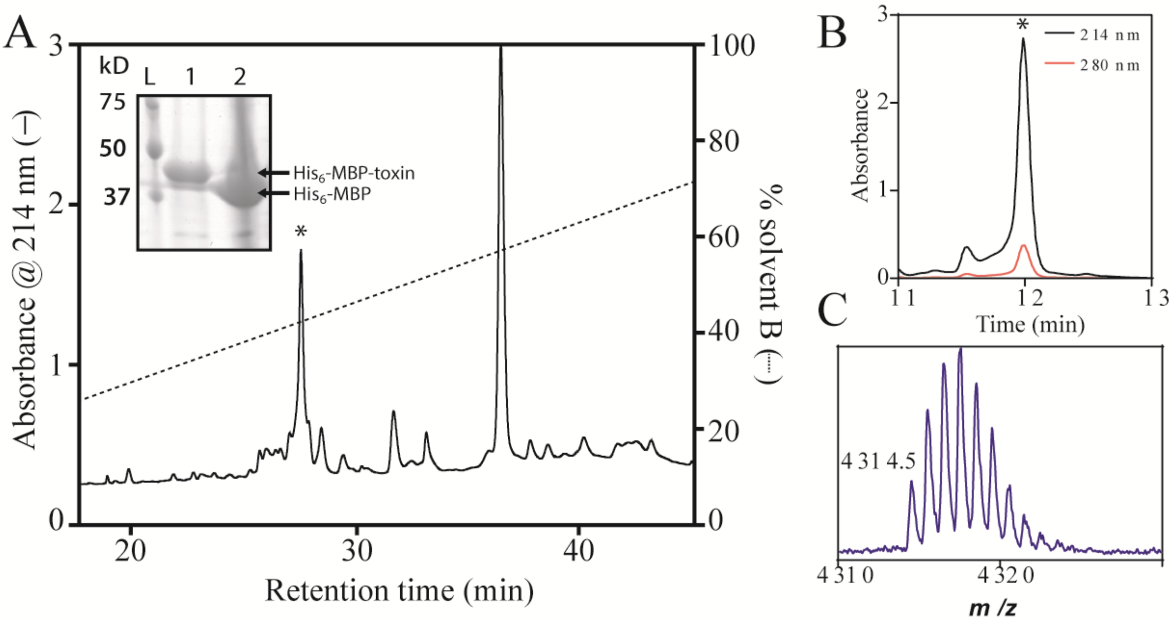

Supplementary Figure 2. Bacterial recombinant expression of Pre1a. **A)** RP-HPLC of cleaved Pre1a from His_6-MBP. SDS-PAGE of His_6-MBP-Pre1a before cleavage (Lane 1) and after cleavage (Lane 2). **B)** Secondary purification of cleaved Pre1a, **C)** resulting in a mass of 4314.5. Theoretical M/Z with oxidised cysteines is 4314.9 (Expasy, Peptide Mass).

A

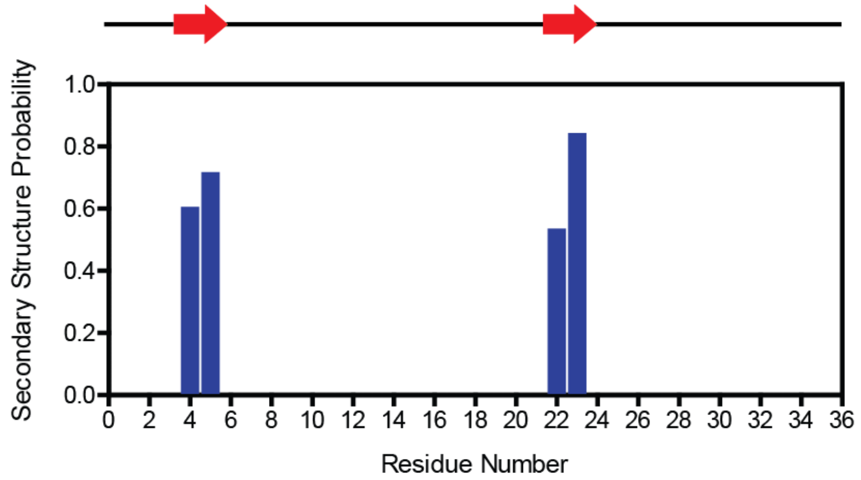

B

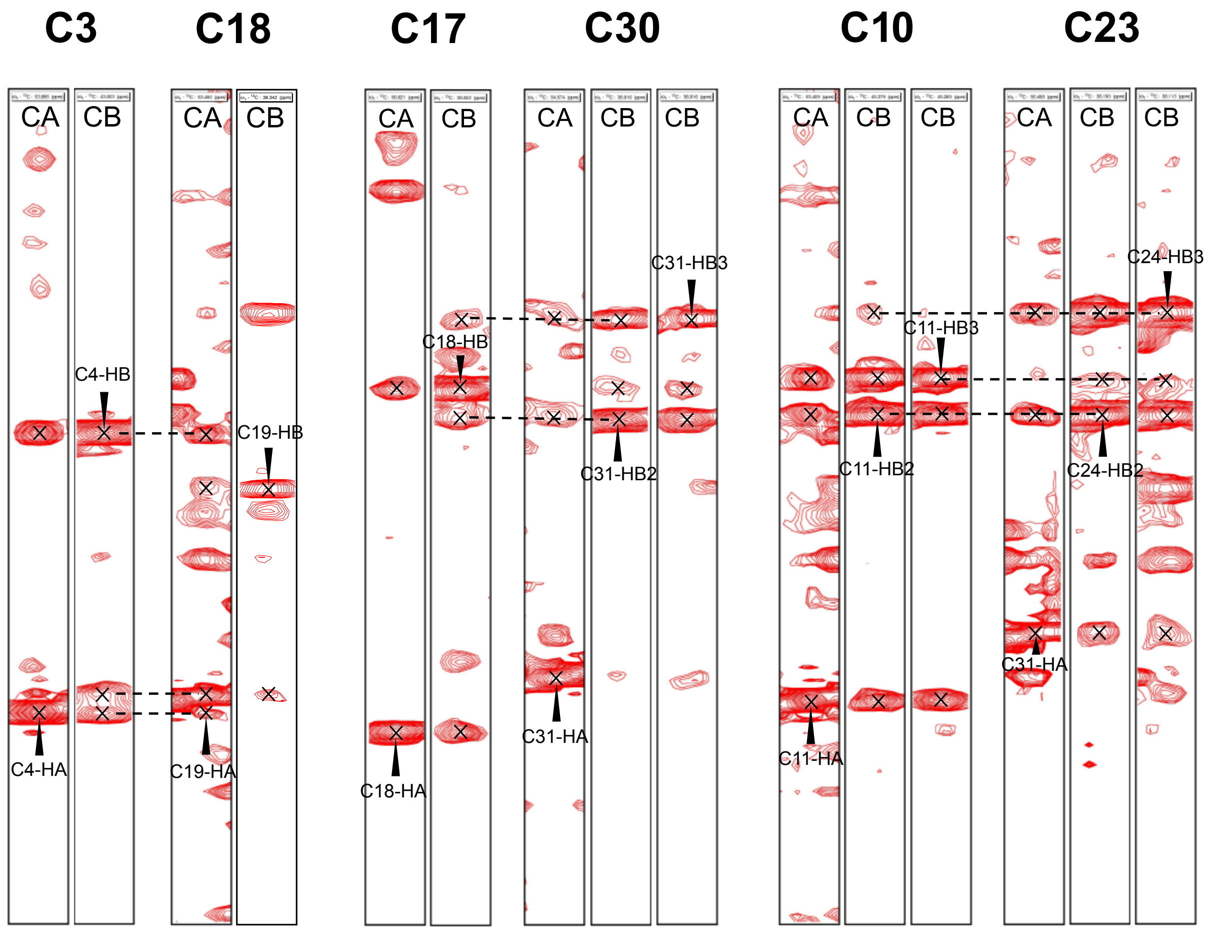

Supplementary Figure 3. A) Secondary structure predictions of rPre1a as calculated by TALOS+. β-sheet predictions are denoted by red arrows. **B)** ^{13}C-NOESY spectra for the six Cys residues in Pre1a showing proximity between C3 and C18, C10 and C23 and C17 and C30, consistent with the disulfide connectivity of the inhibitor cystine knot motif (C1–C4, C2–C5, C3–C6).

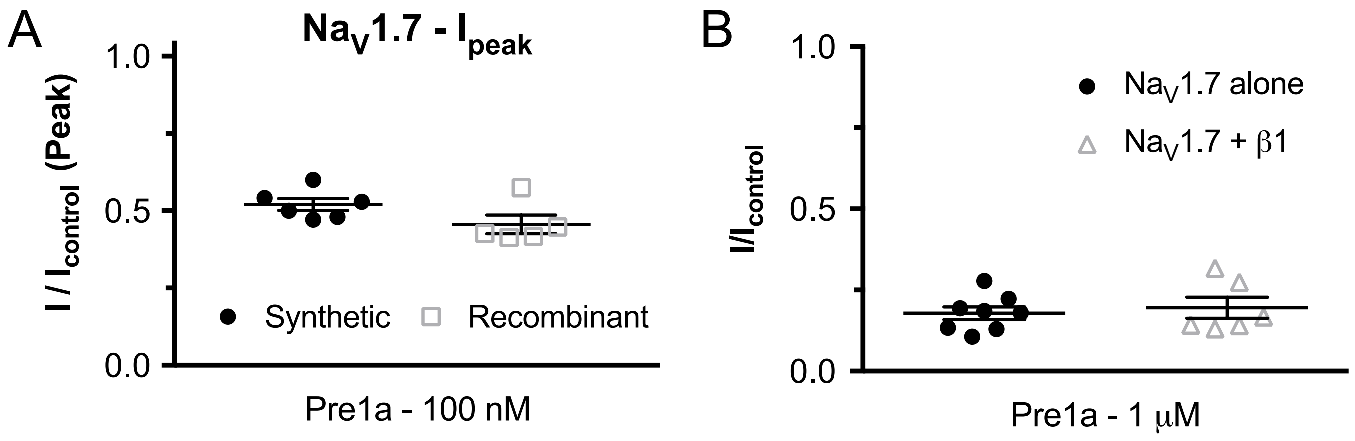

Supplementary Figure 4. (A) Comparison of the effect of 100 nM synthetic and recombinant Pre1a on the peak current of hNa$_V$1.7 expressed in *Xenopus* oocytes. The inhibitory activity is the same for peptide produced by either means. (n=5-6, P=0.0968, unpaired t test). (B) Comparison of the effect of 1 μM recombinant Pre1a on the peak current of hNa$_V$1.7 α-subunit alone and co-expressed with the β1 accessory subunit (expressed in *Xenopus* oocytes). There is no statistical difference in the effect of Pre1a on Na$_V$1.7 in the presence or absence of the β1 subunit (n=7-8, P=0.6561, unpaired t test).
